# Pyridine indole hybrids as novel potent CYP17A1 inhibitors

**DOI:** 10.1080/14756366.2025.2463014

**Published:** 2025-02-14

**Authors:** Tomasz M. Wróbel, Angelika Grudzińska, Jibira Yakubu, Therina du Toit, Katyayani Sharma, Jeremiah C. Harrington, Fredrik Björkling, Flemming Steen Jørgensen, Amit V. Pandey

**Affiliations:** ^a^ Department of Synthesis and Chemical Technology of Pharmaceutical Substances, Medical University of Lublin, Lublin, Poland; ^b^ Department of Drug Design and Pharmacology, University of Copenhagen, Copenhagen, Denmark; ^c^ Pediatric Endocrinology, Department of Pediatrics, University Children’s Hospital, University of Bern, Bern, Switzerland; ^d^ Translational Hormone Research Program, Department of Biomedical Research, University of Bern, Bern, Switzerland; ^e^ Graduate School for Cellular and Biomedical Sciences, University of Bern, Bern, Switzerland; ^f^ Department of Nephrology and Hypertension, University Hospital Bern, Inselspital, University of Bern, Bern, Switzerland

**Keywords:** CYP17A1, enzyme inhibition, prostate cancer, inhibitors

## Abstract

Prostate cancer (PCa) is one of the most prevalent malignancies affecting men worldwide, and androgen deprivation therapy (ADT) is a primary treatment approach. CYP17A1 inhibitors like abiraterone target the steroidogenic pathway to reduce androgen levels, but their clinical efficacy is limited by drug resistance and adverse effects. This study reports the synthesis and evaluation of novel CYP17A1 inhibitors derived from a previously identified hit compound. Several analogs were synthesised, including an unexpected di-cyano derivative, which demonstrated increased potency against CYP17A1 compared to abiraterone. Biological assays revealed that these compounds significantly inhibited CYP17A1 enzymatic activity and altered steroid biosynthesis. Among the newly synthesised inhibitors, compound **11** showed the highest potency (IC_50_ = 4 nM) and the related compound **14** presented a template for further development. A combined docking and molecular dynamics approach was used to identify the possible target binding modes of the compounds.

## GRAPHICAL ABSTRACT

**Figure:**
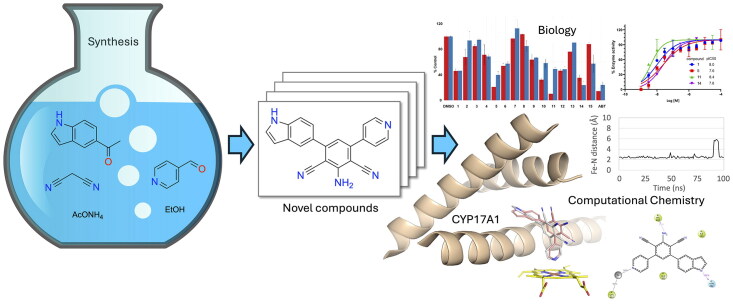

## Introduction

**Figure 1.**
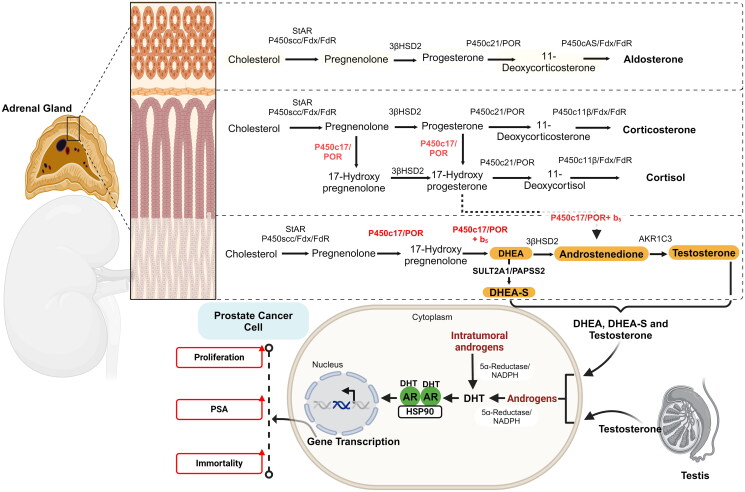
Adrenal steroidogenesis. The steroidogenic pathway in the adrenal cortex as shown, highlights the roles of the different zones: the *Zona glomerulosa*, the outermost layer, responsible for synthesising aldosterone, a mineralocorticoid: the *Zona fasciculata*, middle of the adrenal cortex, this zone primarily produces glucocorticoids, such as cortisol. The zona fasciculata is regulated by the hypothalamic-pituitary-adrenal (HPA) axis via the secretion of adrenocorticotropic hormone (ACTH) from the pituitary. The *Zona reticularis*, the innermost layer of the adrenal cortex is responsible for the production of adrenal androgens, including dehydroepiandrosterone (DHEA), DHEA sulphate (DHEA-S), and testosterone. These hormones can be converted into more potent androgens, dihydrotestosterone (DHT) or oestrogens in peripheral tissues. While not regulated directly by the HPA axis, adrenal androgen production is influenced by ACTH to a lesser extent than glucocorticoids. In prostate cancer, adrenal androgens—along with those produced by the testes—are converted to DHT. DHT binds to androgen receptor, mediates gene transcription, and leads to cancer cell proliferation, increasing PSA (Prostate-Specific Antigen) levels, and contributing to the development of cancer cell immortality. Steroidogenesis involves several key enzymes, including P450scc (Cholesterol side-chain cleavage enzyme, CYP11A1), P450c17 (17α-hydroxylase/17,20-lyase, CYP17A1), P450c21 (21-hydroxylase, CYP21A2), P450cAS (Aldosterone synthase, CYP11B2), P450c11 (11β-hydroxylase, CYP11B1), 3β-HSD (3β-hydroxysteroid dehydrogenase), SULT2A1 (Sulfotransferase), AKR1C3 (Aldo-keto reductase family 1, member C3), and cholesterol transport protein StAR (Steroidogenic acute regulatory protein).

Prostate cancer (PCa) ranks as the second most prevalent malignancy among men globally^1^. Tumour progression is promoted by androgens, with testosterone and dihydrotestosterone exerting pronounced influence on disease advancement. Consequently, androgen deprivation therapy (ADT) has become a standard treatment of prostate cancer. However, various strategies aimed at lowering androgen levels encounter limitations, primarily attributed to the inevitable progression of the disease to the castration-resistant prostate cancer (CRPC)^2^. The main cause of CRPC is persistent androgen receptor signalling. A widely used therapeutic approach involves androgen receptor (AR) antagonists, such as enzalutamide (MDV3100) or apalutamide or darolutamide (ODM-201)^3^. An alternative approach to limit AR stimulation relies on inhibition of the enzymes in the steroidogenic pathway. This treatment modality is represented by abiraterone (CB7598), which is currently the only inhibitor of the cytochrome P450 17α-hydroxylase/17,20-lyase (CYP17A1) enzyme approved by FDA and EMA^4^. The CYP17A1 is a microsomal enzyme that catalyses two discrete biochemical processes on steroids: 17α-hydroxylation and 17,20-lyase conversion (Figure 1). These CYP17A1 catalysed reactions play a crucial role in the intricate process of steroid biosynthesis originating from both the adrenal and gonadal sources^5^. Given its capacity to catalyse steroid transformation reactions through both conventional and alternative pathways^6^, particularly when activated in castration-resistant prostate cancer (CRPC), CYP17A1 has emerged as a promising therapeutic target for the treatment of advanced stages of prostate cancer^7^^,^^8^.

**Figure 2.**
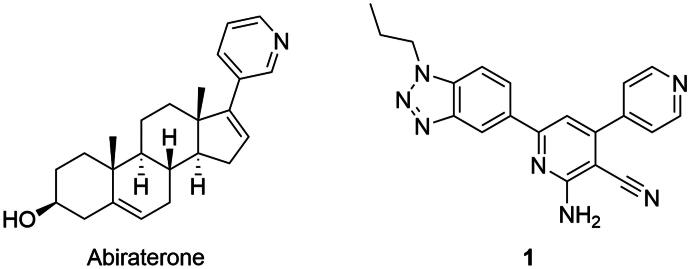
Structures of abiraterone and hit compound **1**.

Abiraterone (Figure 2) comprises a steroid scaffold akin to the endogenous substrates of CYP17A1. However, abiraterone use may give rise to undesired drug interactions and side effects, such as hepatotoxicity. Moreover, the non-selective action of abiraterone contributes to side effects associated with mineralocorticoid metabolism disorders, through the regulation of the mineralocorticoid and glucocorticoid biosynthetic pathway, including inhibition of CYP21A2 activity^9^. In addition, abiraterone metabolites can act as AR agonists promoting prostate cancer progression^10^^,^^11^. The limited efficacy of abiraterone treatment due to emerging drug resistance underscores the importance of the search for improved CYP17A1 inhibitors, with the goal of augmenting treatment efficacy and minimising side effects^12^^,^^13^.

Numerous CYP17A1 inhibitors have been developed over the years and several of them have reached clinical trials^14–16^. These studies provide invaluable insights for the design and investigation of new CYP17A1 inhibitors. Prevailing literature underscores the importance of a coordinated binding between the heteroatom of the ligand and the haem iron in the enzyme active site as the pivotal interaction in CYP17A1 inhibition^17^. Substantial evidence suggests the superior efficacy of ligands containing heteroaromatic fragments, such as pyridine and benzimidazole^18^^,^^19^.

In our present work, we followed up on the identified hit compound **1** (Figure 2) from our previous screening campaign^18^. Several analogs were envisioned to explore structure-activity relationship (SAR). During the synthesis, an unexpected outcome occurred leading to compounds with markedly increased activity compared to those of the initial design. Here we describe synthesis, biological activity, and computational studies on newly synthesised CYP17A1 inhibitors.

## Materials and methods

### General chemistry information

^1^H NMR and ^13^C NMR spectra were recorded on Bruker 400 MHz and 600 MHz Avance III spectrometer equipped with a BBO probe. Spectra are referenced to the residual solvent peak or internal standard (TMS). HRMS spectra were acquired on Thermo QExactive Orbitrap Plus mass spectrometer coupled with Thermo Vanquish UHPLC. Samples in 33% methanol (injection volume 5 µL) were measured using the full scan filter (200–500 m/z) in positive ion mode (ESI+). Reagents were obtained from commercial sources and used as received unless stated otherwise. Solvents were used as received or purified and dried by standard methods when necessary. Reactions yields were unoptimised.

### Synthesis of compounds 1–15

#### 2-Amino-6–(1-propyl-1H-benzo[d][1,2,3]triazol-5-yl)-[4,4′-bipyridine]-3-carbonitrile (1)

Isonicotinaldehyde (107 mg, 1 mmol), malononitrile (66 mg, 1 mmol), and ammonium acetate (77 g, 1 mmol) were added to a flask with refluxing absolute EtOH (to a 0.2 mmol/L concentration). Simultaneously, 1–(1-propyl-1*H*-benzo[*d*][1,2,3]triazol-5-yl)ethan-1-one (203 mg, 1 mmol) and ammonium acetate (77 g, 1 mmol) were added to another flask with refluxing absolute EtOH (to a 0.2 mmol/L concentration). Both flasks were allowed to reflux for 12 min before combining. The combined mixture was refluxed for 24 h. The volatiles were removed on a rotavap and the remaining residue was purified by flash column chromatography on silica gel and subsequently by preparative HPLC to yield a light yellow/orange liquid (20 mg, 6%).

^1^H NMR (600 MHz, DMSO) δ 8.93 (d, *J* = 5.1 Hz, 2H), 8.75 (s, 1H), 8.25 (dd, *J* = 9.1, 1.6 Hz, 1H), 8.03 (d, *J* = 9.0 Hz, 1H), 8.01–7.95 (m, 2H), 7.57 (s, 1H), 7.27 (s, 2H), 4.75 (t, *J* = 6.9 Hz, 2H), 2.07 (h, *J* = 7.2 Hz, 2H), 0.89 (t, *J* = 7.4 Hz, 3H).

^13^C NMR (151 MHz, DMSO) δ 161.2, 159.3, 151.9, 148.0, 147.8, 145.0, 144.4, 135.9, 126.0, 124.8, 118.4, 117.9, 116.8, 109.9, 86.5, 58.2, 23.4, 11.4.

HMRS (ESI) [M + H]^+^ calc. 356.1618 found 356.1614.

#### 2-Amino-4,6-diphenylnicotinonitrile (2)

Benzaldehyde (106 mg, 1 mmol), acetophenone (120 mg, 1 mmol), malononitrile (66 mg, 1 mmol), and ammonium acetate (616 mg, 8 mmol) were refluxed in EtOH for 45 min. The reaction mixture was allowed to cool to room temperature and the formed precipitate was filtered and washed with ice cold ethanol. The formed precipitate was purified by flash column chromatography on silica gel eluting with DCM-MeOH, 0–5% gradient and subsequently recrystallized from ethyl acetate/heptane to yield a white solid (89 mg, 33%).

^1^H NMR (600 MHz, DMSO) δ 8.16–8.11 (m, 2H), 7.71–7.67 (m, 2H), 7.59–7.54 (m, 3H), 7.52–7.47 (m, 3H), 7.28 (s, 1H), 7.01 (s, 2H).

^13^C NMR (151 MHz, DMSO) δ 161.3, 159.1, 155.4, 138.0, 137.5, 130.6, 130.1, 129.2, 129.1, 128.8, 127.7, 117.5, 109.7, 87.1.

HMRS (ESI) [M + H]^+^ calc. 272.1182 found 272.1176.

#### 6-Amino-4-phenyl-[2,3′-bipyridine]-5-carbonitrile (3)

Benzaldehyde (106 mg, 1 mmol), 3-acetylpyridine (121 mg, 1 mmol), malononitrile (66 mg, 1 mmol), and ammonium acetate (616 mg, 8 mmol) were refluxed in EtOH for 45 min. The volatiles were removed on a rotavap and the remaining residue was purified by flash column chromatography on silica gel eluting with DCM-MeOH, 0–5% gradient, and subsequently by preparative HPLC to yield a light orange liquid (60 mg, 22%).

^1^H NMR (600 MHz, DMSO) δ 9.42 (d, *J* = 2.0 Hz, 1H), 8.87–8.81 (m, 2H), 7.88–7.84 (m, 1H), 7.73–7.69 (m, 2H), 7.61–7.55 (m, 3H), 7.50 (s, 1H), 7.20 (s, 2H).

^13^C NMR (151 MHz, DMSO) δ 161.3, 156.0, 155.0, 147.3, 145.3, 139.2, 137.0, 135.1, 130.3, 129.2, 128.9, 125.8, 117.1, 110.3, 88.7.

HMRS (ESI) [M + H]^+^ calc. 273.1135 found 273.1129.

#### 2′-Amino-6′-phenyl-[3,4′-bipyridine]-3′-carbonitrile (4)

Nicotinaldehyde (107 mg, 1 mmol), acetophenone (120 mg, 1 mmol), malononitrile (66 mg, 1 mmol), and ammonium acetate (616 mg, 8 mmol) were refluxed in EtOH for 45 min. The volatiles were removed on a rotavap and the remaining residue was purified by flash column chromatography on silica gel eluting with DCM-MeOH, 0–5% gradient, and subsequently by preparative HPLC to yield a light orange liquid (44 mg, 16%).

^1^H NMR (600 MHz, DMSO) δ 9.00 (dd, *J* = 2.3, 0.8 Hz, 1H), 8.83 (dd, *J* = 5.0, 1.6 Hz, 1H), 8.34 (ddd, *J* = 7.9, 2.3, 1.6 Hz, 1H), 8.18–8.13 (m, 2H), 7.78 (ddd, *J* = 7.9, 5.1, 0.8 Hz, 1H), 7.51 (tdd, *J* = 4.7, 3.6, 1.5 Hz, 3H), 7.42 (s, 1H), 7.17 (s, 2H).

^13^C NMR (151 MHz, DMSO) δ 161.2, 159.4, 151.3, 149.0, 147.4, 139.0, 137.8, 134.1, 130.8, 129.1, 127.8, 125.0, 117.1, 109.9, 87.1.

HMRS (ESI) [M + H]^+^ calc. 273.1135 found 273.1129.

#### 2-Amino-6-(1H-indol-5-yl)-[4,4′-bipyridine]-3-carbonitrile (5)

Isonicotinaldehyde (107 mg, 1 mmol), malononitrile (66 mg, 1 mmol), and ammonium acetate (77 g, 1 mmol) were added to a flask with refluxing absolute EtOH (to a 0.2 mmol/L concentration). Simultaneously, 1–(1-propyl-1*H*-benzo[*d*][1,2,3]triazol-5-yl)ethan-1-one (203 mg, 1 mmol) and ammonium acetate (77 mg, 1 mmol) were added to another flask with refluxing absolute EtOH (to a 0.2 mmol/L concentration). Both flasks were allowed to reflux for 12 min before combining. The combined mixture was refluxed for 24 h. The reaction mixture was filtered and the volatiles were removed on a rotavap and the remaining residue was purified by flash column chromatography on silica gel and subsequently by preparative HPLC to yield a light yellow/orange solid (12 mg, 4%).

^1^H NMR (600 MHz, DMSO) δ 11.33 (s, 1H), 8.95–8.92 (m, 2H), 8.43 (d, *J* = 1.7 Hz, 1H), 8.02–7.98 (m, 2H), 7.95 (dd, *J* = 8.6, 1.8 Hz, 1H), 7.48 (dt, *J* = 8.6, 0.8 Hz, 1H), 7.42 (dd, *J* = 3.1, 2.3 Hz, 1H), 7.40 (s, 1H), 7.13 (s, 2H), 6.54 (ddd, *J* = 3.0, 1.9, 0.9 Hz, 1H).

^13^C NMR (151 MHz, DMSO) δ 161.3, 161.2, 151.2, 148.7, 147.5, 137.8, 128.6, 128.3, 127.2, 125.0, 121.2, 120.6, 117.1, 112.0, 109.0, 102.7, 84.7.

HMRS (ESI) [M + H]^+^ calc. 312.1244 found 312.1237.

#### 3-Amino-5-(pyridin-4-yl)-[1,1′-biphenyl]-2,4-dicarbonitrile (6)

Isonicotinaldehyde (107 mg, 1 mmol), acetophenone (120 mg, 1 mmol), malononitrile (66 mg, 1 mmol), and ammonium acetate (616 mg, 8 mmol) were refluxed in EtOH for 45 min. The reaction mixture was allowed to cool to room temperature and the formed precipitate was filtered and washed with ice cold ethanol. The formed precipitate was purified by flash column chromatography on silica gel eluting with DCM-MeOH, 0–5% gradient and subsequently recrystallized from ethyl acetate to yield a light-yellow solid (92 mg, 31%).

^1^H NMR (600 MHz, DMSO) δ 8.77–8.72 (m, 2H), 7.68–7.62 (m, 4H), 7.57–7.50 (m, 3H), 6.95 (s, 2H), 6.87 (s, 1H).

^13^C NMR (151 MHz, DMSO) δ 154.5, 150.7, 150.5, 147.6, 145.3, 137.7, 130.1, 129.2, 129.1, 123.7, 118.7, 116.3, 116.0, 95.7, 94.2.

HMRS (ESI) [M + H]^+^ calc. 297.1135 found 297.1134.

#### 2-Amino-4,6-di(pyridin-3-yl)isophthalonitrile (7)

Nicotinaldehyde (107 mg, 1 mmol), 3-acetylpyridine (121 mg, 1 mmol), malononitrile (66 mg, 1 mmol), and ammonium acetate (616 mg, 8 mmol) were refluxed in EtOH for 45 min. The reaction mixture was allowed to cool to room temperature and the formed precipitate was filtered and washed with ice cold ethanol. The formed precipitate was purified by flash column chromatography on silica gel eluting with DCM-MeOH, 0–5% gradient and subsequently recrystallized from ethyl acetate to yield a beige-yellow solid (83 mg, 28%).

^1^H NMR (600 MHz, DMSO) δ 8.85 (d, *J* = 2.1 Hz, 2H), 8.74–8.69 (m, 2H), 8.13–8.07 (m, 2H), 7.58 (dd, *J* = 7.9, 4.9 Hz, 2H), 7.02 (s, 2H), 7.00 (s, 1H).

^13^C NMR (151 MHz, DMSO) δ 154.5, 150.8, 149.3, 147.3, 136.8, 133.7, 124.0, 119.1, 116.2, 95.3.

HMRS (ESI) [M + H]^+^ calc. 298.1087 found 297.1085.

#### 2-Amino-4,6-di(pyridin-4-yl)isophthalonitrile (8)

Isonicotinaldehyde (107 mg, 1 mmol), 4-acetylpyridine (121 mg, 1 mmol), malononitrile (66 mg, 1 mmol), and ammonium acetate (616 mg, 8 mmol) were refluxed in EtOH for 45 min. The reaction mixture was allowed to cool to room temperature and the formed precipitate was filtered and washed with ice cold ethanol. The formed precipitate was purified by flash column chromatography on silica gel eluting with DCM-MeOH, 0–5% gradient, and subsequently by preparative HPLC to yield an off-white solid (77 mg, 26%).

^1^H NMR (600 MHz, DMSO) δ 8.81–8.70 (m, 4H), 7.72–7.61 (m, 4H), 7.09 (s, 2H), 6.94 (s, 1H).

^13^C NMR (151 MHz, DMSO) δ 154.5, 150.5, 147.9, 145.1, 123.7, 118.4, 115.8, 95.3.

HMRS (ESI) [M + H]^+^ calc. 298.1087 found 298.1084.

#### 2-Amino-4-(pyridin-3-yl)-6-(pyridin-4-yl)isophthalonitrile (9)

Nicotinaldehyde (107 mg, 1 mmol), 4-acetylpyridine (121 mg, 1 mmol), malononitrile (66 mg, 1 mmol), and ammonium acetate (616 mg, 8 mmol) were refluxed in EtOH for 45 min. The reaction mixture was allowed to cool to room temperature and the formed precipitate was filtered and washed with ice cold ethanol. The formed precipitate was purified by flash column chromatography on silica gel eluting with DCM-MeOH, 0–5% gradient, and subsequently by preparative HPLC to yield an off-white solid (104 mg, 35%).

^1^H NMR (600 MHz, DMSO) δ 8.89 (d, *J* = 2.3 Hz, 1H), 8.83 (d, *J* = 5.3 Hz, 2H), 8.75 (dd, *J* = 4.9, 1.6 Hz, 1H), 8.21–8.16 (m, 1H), 7.85–7.79 (m, 2H), 7.68–7.62 (m, 1H), 7.11 (s, 2H), 7.01 (s, 1H).

^13^C NMR (151 MHz, DMSO) δ 154.5, 150.3, 149.1, 148.7, 147.3, 147.2, 146.8, 137.6, 133.8, 124.4, 124.3, 118.8, 116.0, 96.1.

HMRS (ESI) [M + H]^+^ calc. 298.1087 found 298.1084.

#### 2-Amino-4–(1-propyl-1H-benzo[d][1,2,3]triazol-5-yl)-6-(pyridin-4-yl)isophthalonitrile (10)

The compound was isolated as the second product from the reaction that produced **1** as a bright yellow solid (27 mg, 7%).

^1^H NMR (600 MHz, DMSO) δ 8.84 (d, *J* = 5.1 Hz, 2H), 8.26 (dd, *J* = 1.7, 0.9 Hz, 1H), 8.08 (dd, *J* = 8.8, 0.9 Hz, 1H), 7.87–7.83 (m, 2H), 7.67 (dd, *J* = 8.8, 1.6 Hz, 1H), 7.05 (s, 2H), 7.02 (s, 1H), 4.77 (t, *J* = 6.9 Hz, 2H), 2.08 (h, *J* = 7.2 Hz, 2H), 0.89 (t, *J* = 7.4 Hz, 3H).

^13^C NMR (151 MHz, DMSO) δ 154.5, 150.5, 148.8, 147.2, 146.9, 144.2, 143.9, 135.7, 127.5, 124.5, 119.1, 119.0, 118.7, 116.2, 115.9, 96.3, 94.3, 58.3, 23.4, 11.4.

HMRS (ESI) [M + H]^+^ calc. 380.1618 found 380.1614.

#### 2-Amino-4-(1H-indol-5-yl)-6-(pyridin-4-yl)isophthalonitrile (11)

The compound was isolated as the second product from the reaction that produced **5** as a bright yellow solid (94 mg, 28%).

^1^H NMR (600 MHz, DMSO) δ 11.36 (s, 1H), 8.78–8.70 (m, 2H), 7.86 (d, *J* = 1.8 Hz, 1H), 7.68–7.65 (m, 2H), 7.53 (dt, *J* = 8.4, 0.8 Hz, 1H), 7.46 (t, *J* = 2.7 Hz, 1H), 7.37 (dd, *J* = 8.5, 1.8 Hz, 1H), 6.89 (s, 1H), 6.85 (s, 2H), 6.54 (ddd, *J* = 3.0, 1.9, 0.9 Hz, 1H).

^13^C NMR (151 MHz, DMSO) δ 154.7, 152.5, 150.5, 147.1, 145.5, 136.9, 128.5, 128.1, 127.3, 123.7, 122.1, 121.3, 119.1, 116.8, 116.3, 112.1, 102.3, 95.8, 93.0.

HMRS (ESI) [M + H]^+^ calc. 336.1244 found 336.1240.

#### 5-(3-(Pyridin-3-yl)phenyl)-1H-indole (12)

Suspension of palladium diacetate (22.3 mg, 99.3 µmol), SPhos (100 mg, 244 µmol) and potassium phosphate (602 mg, 2.84 mmol) in dioxane were added to the solution of (1H-indol-5-yl)boronic acid (457 mg, 2.84 mmol) and 3-(3 bromophenyl)pyridine (0.332 mg, 1.42 mmol) in dioxane/water (3:1) under inert atmosphere. The reaction mixture was heated at 100 °C for 1.5 h under microwave radiation. The reaction mixture was allowed to reach room temperature and the solvent was evaporated. The residue was purified by dry column vacuum chromatography (eluent: ethyl acetate/heptane 80:20 v/v) to yield oily residue which was triturated with diethyl ether to give white solid (57 mg, 15%).

^1^H NMR (600 MHz, DMSO) δ 11.20 (s, 1H), 9.01 (s, 1H), 8.61 (s, 1H), 8.19 (d, *J* = 6.5 Hz, 1H), 7.97 (d, *J* = 7.2 Hz, 2H), 7.77–7.39 (m, 9H), 6.52 (s, 1H).

^13^C NMR (151 MHz, DMSO) δ 148.9, 148.3, 143.3, 138.1, 136.4, 136.1, 134.9, 131.5, 130.1, 128.8, 127.0, 126.6, 125.8, 125.2, 124.3, 121.0, 119.0, 112.3, 102.1.

HMRS (ESI) [M + H]^+^ calc. 271.1230 found 271.1230.

#### 5-(4-(Pyridin-3-yl)phenyl)-1H-indole (13)

Suspension of palladium diacetate (47 mg, 3 mmol), SPhos (1.4 g, 3.3 mmol) and potassium phosphate (1.3 g, 6 mmol) in dioxane were added to the solution of (1H-indol-5-yl)boronic acid (970 mg, 6 mmol) and 3-(4-bromophenyl)pyridine (700 mg, 3 mmol) in dioxane/water (3:1) under inert atmosphere. The reaction mixture was stirred at reflux overnight, then it was allowed to reach room temperature and it was filtered to give a white solid. It was washed with methanol to yield the title compound as a white solid (551 mg, 68%).

^1^H NMR (600 MHz, DMSO) δ 11.19 (s, 1H), 8.98–8.94 (m, 1H), 8.58 (d, *J* = 3.9 Hz, 1H), 8.14 (d, *J* = 7.9 Hz, 1H), 7.90 (s, 1H), 7.81 (s, 4H), 7.53–7.46 (m, 3H), 7.41 (d, *J* = 2.5 Hz, 1H), 6.52 (s, 1H).

^13^C NMR (151 MHz, DMSO) δ 148.8, 148.0, 142.1, 136.1, 135.7, 135.2, 134.3, 131.0, 128.8, 127.7, 126.7, 124.4, 120.7, 118.6, 112.3, 102.1, 66.8.

HMRS (ESI) [M + H]^+^ calc. 271.1230 found 271.1230.

#### 5-(3-(Pyridin-4-yl)phenyl)-1H-indole (14)

4-(3-Bromophenyl)pyridine (0.2 g 1 mmol), (1*H*-indol-5-yl)boronic acid (0.3 g, 2 mmol), Tetrakis(triphenylphosphine)palladium(0) (0.1 g, 0.1 mmol) and potassium carbonate (3 mmol) were added to the mixture of dioxane and water 2:1 under inert atmosphere. The reaction mixture was stirred at 65 °C overnight, then it was allowed to reach room temperature and it was filtered. The filtrate was evaporated providing yellow oil. The crude was purified by dry column vacuum chromatography (eluent: ethyl acetate/dcm 50:50 v/v) to give white solid. The solid was washed by methanol to yield the titled compound as a white solid (60 mg, 20%).

^1^H NMR (600 MHz, DMSO) δ 11.19 (s, 1H), 8.68–8.66 (m, 2H), 8.04 (t, *J* = 1.7 Hz, 1H), 7.97–7.96 (m, 1H), 7.84–7.82 (m, 2H), 7.78 (ddd, *J* = 7.7, 1.7, 1.1 Hz, 1H), 7.72 (ddd, *J* = 7.7, 1.8, 1.0 Hz, 1H), 7.52–7.50 (m, 2H), 7.42–7.40 (m, 1H), 6.52–6.51 (m, 1H).

^13^C NMR (151 MHz, DMSO) δ 150.7, 147.8, 143.4, 138.3, 136.1, 131.3, 130.2, 128.8, 128.0, 126.7, 125.6, 125.1, 122.0, 121.0, 119.0, 112.3, 102.1.

HMRS (ESI) [M + H]^+^ calc. 271.1230 found 271.1230.

#### 5-(4-(Pyridin-4-yl)phenyl)-1H-indole (15)

Suspension of palladium diacetate (30 mg, 0.1 mmol), XPhos (90 mg, 0.2 mmol) and potassium phosphate (800 mg, 4 mmol) in dioxane were added to the solution of (1H-indol-5-yl)boronic acid (600 mg, 4 mmol) and 4-(4-bromophenyl)pyridine (500 mg, 2 mmol) in dioxane/water (3:1) under inert atmosphere. The reaction mixture was stirred at reflux overnight, then it was allowed to reach room temperature and it was filtered to give a white solid. It was washed with methanol to yield the title compound as a white solid (250 mg, 50%).

^1^H NMR (600 MHz, DMSO) δ 11.20 (s, 1H), 8.65 (dd, *J* = 4.6, 1.4 Hz, 2H), 7.94–7.87 (m, 3H), 7.83 (d, *J* = 8.4 Hz, 2H), 7.80–7.75 (m, 2H), 7.53–7.46 (m, 2H), 7.40 (t, *J* = 2.7 Hz, 1H), 6.52 (s, 1H).

^13^C NMR (151 MHz, DMSO) δ 150.1, 146.5, 142.7, 135.6, 134.6, 130.2, 128.2, 127.2, 127.1, 126.1, 120.8, 120.2, 118.2, 111.8, 101.5.

HMRS (ESI) [M + H]^+^ calc. 271.1230 found 271.1229.

### General biology information

The 3 beta-hydroxysteroid dehydrogenases inhibitor, trilostane was isolated from Modrenal^®^ tablets obtained from Bioenvision in New York, USA using absolute ethanol^20^. Abiraterone acetate was acquired from MedChemExpress through Lucerna Chem AG, based in Lucerne, Switzerland. Radiolabelled compounds included Progesterone [4-14C] (specific activity: 55 mCi/mmol; concentration: 0.1 mCi/mL), 17α-Hydroxypregnenolone [21-3H] (specific activity: 15 Ci/mmol; concentration: 1 mCi/mL), and [3H]-17α-OH progesterone [1,2,6,7-3H] (specific activity: 60–120 Ci/mmol; 2.22–4.44 TBq/mmol) and were sourced from American Radiolabelled Chemicals Inc., St. Louis, MO, USA. Non-radioactive compounds, such as Pregnenolone, progesterone, 17α-hydroxyprogesterone, 17α-hydroxypregnenolone, Resazurin sodium salt, and Dimethyl sulfoxide (DMSO) were procured from Sigma-Aldrich, St. Louis, MO, USA. Organic solvents were obtained from Carl Roth^®^ GmbH + Co. KG, Karlsruhe, Germany, and activated charcoal from Merck AG, Darmstadt, Germany. Silica gel-coated aluminium-backed TLC plates were purchased from Macherey-Nagel, Oensingen, Switzerland. Tritium screens for autoradiography were provided by Fujifilm, Dielsdorf, Switzerland.

### Cell cultures

Prostate cancer cell lines LNCaP FGC were acquired from the American Type Culture Collection (ATCC: CRL-1740) and maintained in RPMI-1640 medium supplemented with 2 mM L-glutamine, 10 mM HEPES, 1 mM sodium pyruvate, 10% foetal bovine serum (FBS), and 1% penicillin-streptomycin (Gibco™, Thermo Fisher Scientific, Waltham, MA, USA). VCaP (ATCC: CRL-2876) and DU-145 (ATCC: HTB-81) cells were kindly provided by Prof. Mark Rubin from the Department of Biomedical Research (DBMR) at the University of Bern, Switzerland. VCaP and DU-145 cells were grown in Dulbecco’s Modified Eagle Medium (DMEM) with 10% FBS, 1% antibiotic mix (100×), and 1 mM sodium pyruvate. Human adrenocortical NCI-H295R cells (ATCC: CRL-2128) were purchased from ATCC and cultured in DMEM/Ham’s F-12 medium with L-glutamine and 15 mM HEPES (Thermo Fisher Scientific, Waltham, MA, USA), supplemented with 5% NU-I serum (Becton Dickinson, Franklin Lakes, NJ, USA), 0.1% insulin, transferrin, selenium (100 U/mL; Thermo Fisher Scientific, Waltham, MA, USA), and 1% penicillin (100 U/mL; Thermo Fisher Scientific, Waltham, MA, USA), and streptomycin (100 μg/mL; GIBCO). RWPE-1 cells (ATCC: CRL-36O7) were cultured in keratinocyte serum free media (K-SFM) supplemented with 0.05 mg/mL bovine pituitary extract and 5 ng/mL epidermal growth factor and 1% penicillin (100 U/mL; Thermo Fisher Scientific, Waltham, MA, USA), and streptomycin (100 μg/mL; GIBCO). Passage numbers remained below 30 as per standard procedures^9^^,^^20–23^. All cell lines were validated and characterised prior to use to ensure their authenticity and reliability. Routine mycoplasma testing was conducted to confirm that the cells were free from contamination. Additionally, key functional and morphological characteristics were assessed to verify their alignment with previously reported profiles^24^.

### Alamar blue assay

LNCaP and RWPE-1 cells were plated in 96-well plates at 10 000 cells per well and incubated overnight at 37 °C with 5% CO_2_. VCaP cells were plated at 12 000 cells per well under the same conditions. After 24 h, the medium was replaced with fresh medium containing the compounds or abiraterone at a final concentration of 10 µM. Cells were incubated for an additional 24 and 48 h. Cell viability was measured using the Alamar Blue assay. Post-incubation, 0.05 mg/mL Alamar Blue solution in phosphate-buffered saline (PBS) was added to each well. The plates were incubated in the dark at 37 °C for 4 h, and fluorescence was measured at an excitation wavelength of 550 nm and an emission wavelength of 590 nm. Cell viability was calculated relative to control sample treated with DMSO. All experiments were conducted in triplicate.

### Scratch assay

Human prostate cancer DU-145 cells show strong cell proliferation and therefore, are an established model for assay of antiproliferative effects of test compounds. DU-145 cells were plated in 24-well plates at 50 000 cells per well and grown until they reached 85% confluence. To inhibit cell proliferation, cells were treated with 5 µg/mL mitomycin for 2 h. A wound was created using a 10 µL pipette tip. The cells were then exposed to drugs at a concentration of 10 µM, while control wells received DMSO. Wound closure was observed and photographed using an optical microscope after 24 h. Images were analysed using ImageJ software (version 1.54j).

### CYP17A1 activity assays

Human adrenal NCI-H295R cells are an established model for assay of CYP17A1 activities^25–27^. The NCI-H295R cells were seeded in 12-well plates at 500 000 cells per well and incubated overnight. On the following day, 10 µM of test compounds were added to the wells with fresh medium and incubated for 4 h. Abiraterone was used as a reference, and DMSO was included as a control. For assessing CYP17A1 hydroxylase activity, cells were treated with [^14^C]-Progesterone at 10 000 cpm/1 µM per well. Trilostane was added prior to the test compounds and substrate to inhibit 3β-hydroxysteroid dehydrogenase activity so that a clean assay of only CYP17A1 activity could be performed^20^^,^^27^^,^^28^. Radiolabeled steroids were extracted from the media using a mixture of ethyl acetate and isooctane (1:1 v/v) and separated by Thin Layer Chromatography (TLC) on silica gel-coated aluminium plates (Supelco^®^ Analytics, Sigma-Aldrich Chemie GmbH, Taufkirchen, Germany). TLC spots were visualised on a phosphor screen and detected by autoradiography using a Typhoon™ FLA-7000 PhosphorImager (GE Healthcare, Uppsala, Sweden). Radioactivity was quantified with ImageQuant™ TL analysis software (GE Healthcare Europe GmbH, Freiburg, Germany), and enzyme activity was expressed as the percentage of radioactivity incorporated into the product relative to the total radioactivity. For CYP17A1 lyase activity, cells were exposed to 50 000 cpm/1 µM [21-^3^H]-17α-hydroxypregnenolone per well under similar conditions. The tritiated water release assay was used to measure the conversion of 17OH-Pregnenolone to DHEA. Steroids in the media were precipitated using a 5% activated charcoal/0.5% dextran solution. Enzyme activity was evaluated based on the water-soluble tritiated by-product formed in equimolar amounts with DHEA, with radioactivity in the aqueous phase quantified by liquid scintillation counting (MicroBeta2^®^ Plate Counter, PerkinElmer Inc., Waltham, MA, USA). Percent inhibition was calculated relative to control samples.

### Steroid analysis using liquid chromatography–mass spectrometry

Steroid quantification was performed using Liquid Chromatography–Mass Spectrometry (LC-MS), following established protocols to study effect of compounds on adrenal steroidogenesis. Human adrenal NCI-H295R cells were seeded in 12-well plates at 500 000 cells per well and treated with fresh growth medium containing the test compounds for 4 h. Subsequently, 1 µM pregnenolone was added and incubated for an additional 4 h. Steroids were extracted from 500 µL aliquots of cell media, separated, and quantified according to the referenced methodology.

### Data analysis

Data analysis was conducted using R Studio (version 3.6.0+) and GraphPad Prism (GraphPad Software, Inc., San Diego, CA, USA). Data from the mass spectrometer were processed using TraceFinder 4.0 (Thermo Fisher Scientific, Waltham, MA, USA).

### Computational chemistry

The CYP17A1 structure complexed with TOK-001 (PDB 3SWZ, resolution 2.40 Å)^17^ was retrieved from the Protein DataBank^29^. The protein was prepared prior to the docking and molecular dynamics studies by the Protein Preparation Wizard^30^^,^^31^, which add hydrogen atoms, fix bond orders, remove water molecules, optimise protonation and hydrogen-bonds according to pH = 7 and performs a short energy minimisation using the OPLS4 force field with max. RMSD for all atoms on 0.30 Å.

Ligands were constructed in Maestro (version 14.0.134) and subsequently subjected to the Ligand Preparation procedure in Maestro to secure proper protonation at pH = 7^30^^,^^31^. This procedure also performs an energy minimisation of the ligands using the OPLS4 force field^32^.

Docking was performed with the GOLD (Genetic Optimisation for Ligand Binding) program (version 2023.2.0)^33^^,^^34^. Default setup was used except that early termination was turned off and 10 poses were sampled for each docking. The docking radius was set to 15 Å with a centre on the iron atom in the haem group. Docking scores were calculated by the specially developed ChemScore function for haem-containing proteins^35^. For constrained docking the distance between the pyridine nitrogen and the haem iron was constrained to be between 1.5 and 3.0 Å.

Molecular dynamics simulations were performed by the Desmond program (version 7.8.134)^31^. Ligand structures from the docking were neutralised by adding a sodium ion and subsequently embedded in a box of water yielding a system on ∼73.000 atoms comprising 7.500 protein atoms, 40 ligand atoms, and nearly 22.000 water molecules. The system was equilibrated by the step-wise default procedure in Desmond comprising the following key steps: (1) Brownian dynamics at 10 K of the NVT ensemble with non-hydrogens restrained, (2) Simulation of the NVT ensemble using a Berendsen thermostat at 10 K in 12 ps with non-hydrogen solute atoms restrained, (3) Simulation of the NPT ensemble using a Berendsen thermostat and a Berendsen barostat at 10 K and 1 atm in 12 ps with non-hydrogen solute atoms restrained, (4) Simulation of the NPT ensemble using a Berendsen thermostat and a Berendsen barostat at 300 K and 1 atm in 12 ps with non-hydrogen solute atoms restrained, (5) Simulation of the NPT ensemble using a Berendsen thermostat and a Berendsen barostat at 300 K and 1 atm in 24 ps. After the equilibration procedure, the system was simulated as an NPT ensemble for 2 × 100 ns (**1** and **10** with the benzotriazole facing the haem group only run once) and 100 frames were collected for each run for analysis. The stability of the systems was checked by calculating the RMSD values for the protein Calpha atoms and the ligand heavy atoms (not shown) and the distance between relevant nitrogen atoms and the Fe in the haem group.

The Pymol program was used for displaying 3D structures^36^.

## Results and discussion

### Chemistry

Planned alterations of the hit compound **1** involved incorporating benzene and heterocyclic rings in various substitution patterns. An attractive method was chosen to obtain the desired scaffold, utilising a multicomponent reaction comprising aldehyde, ketone, malononitrile, and ammonium acetate^37^.

While the reaction provided the desired compounds, we also noticed the presence of an unexpected new compound with an increased mass of 24 Da (determined by MS) compared to the anticipated compound. This suggested a compound with two additional carbon atoms. Analysis of proton NMR spectra revealed a great deal of similarity between the two compounds. However, carbon NMR spectra of the new compound showed fewer signals in comparison with the target compound. Moreover, the number of signals were further reduced when similar (symmetric) aromatic ketones and aldehydes were used. Neither the original report on the multicomponent synthesis nor the other publications where pyridine containing compounds were used^37^^,^^38^, mentioned the generation of a substantial amount of “+24” compound.

**Scheme 1.**
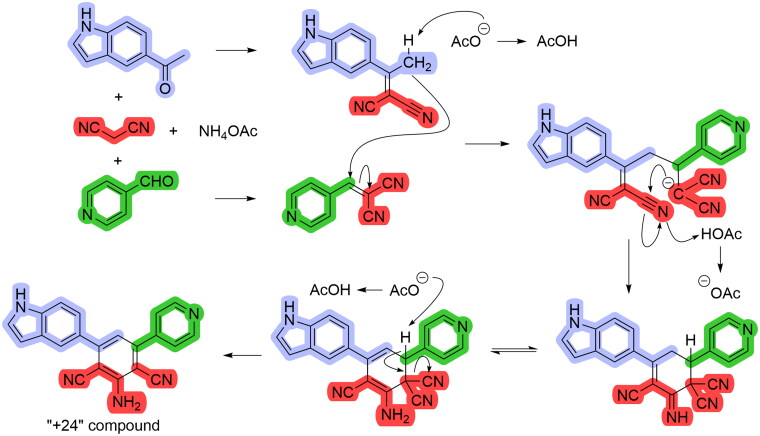
Proposed mechanism of a “+24” compound formation.

Further analysis of the spectral data and literature search on reactions involving possible intermediates^39^^,^^40^ determined the “+24” compound to contain two cyano groups. It forms when both the ketone and aldehyde undergo Knoevenagel condensation with malononitrile, and the resulting products react with one another in Michael reaction. The intermediate produced then cyclizes, with a subsequent loss of one cyano group, leading to the aromatisation of the central ring (Scheme 1). Indeed, such structures bearing two cyano groups align perfectly with the spectral data.

**Scheme 2.**
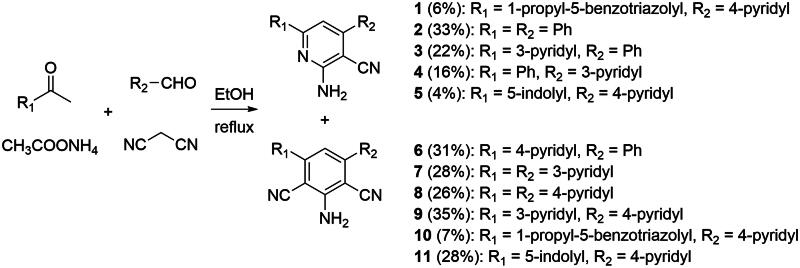
General reaction scheme of the synthesis of compounds **1–11**. Compounds **1**/**10** and **5**/**11** were produced in a single reaction and separated chromatographically. Other compounds were isolated as a single compound. Yields are provided for individual compounds in parentheses.

Consequently, we synthesised ten novel compounds and reproduced compound **1**, originally obtained commercially and found to be a hit during a screening campaign^18^. Both mono-cyano and di-cyano compounds were produced in the same reaction (Scheme 2) and separated chromatographically when possible. We decided to include those “+24” (di-cyano) compounds in our biological screening and interestingly they were found to be potent CYP17A1 inhibitors. Notably, one compound in this series exhibited increased activity compared to abiraterone.

**Scheme 3.**
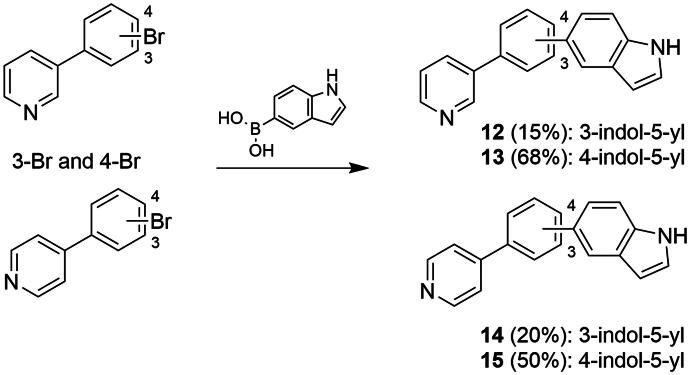
Synthesis of compounds **12–15**. **12** Pd(OAc)_2_, SPhos, K_3_PO_4_, dioxane, reflux; **13** Pd(PPh_3_)_4_, K_2_CO_3_, dioxane, 60 °C, 12 h; **14** Pd(PPh_3_)_4_, K_2_CO_3_, dioxane, 60 °C, 12 h; **15** Pd(OAc)_2_, XPhos, K_3_PO_4_, dioxane, reflux. Yields are provided for individual compounds in parentheses.

Having identified the most potent compound in the first series we were interested in validating its scaffold with further development of possible analogs in mind. Our objective involved simplification of the structures considering geometric patterns and substituent effects. We designed four additional molecules, stripped of substituents and with altered geometry to obtain four distinct core structures. The synthesis, in this case, utilised a common intermediate (1H-indol-5-yl)boronic acid coupled with the corresponding pyridines in a Suzuki reaction (Scheme 3).

### CYP17A1 inhibition

**Figure 3.**
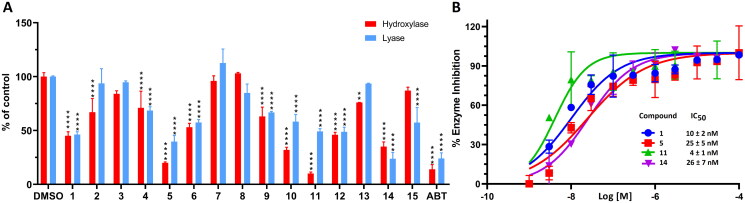
The activity of CYP17A1 evaluated in H295R cells treated with the compounds. (A) shows the impact of the compounds on the 17α-hydroxylase and 17,20-lyase activity, with DMSO as a negative control and abiraterone (ABT) as a positive control. The data indicate the percentage of activity remaining relative to the control. (B) presents the dose-response curves for inhibition of 17,20-lyase activity. Values are presented as mean ± standard deviation from three independent experiments. Statistical significance was determined using [Two-way ANNOVA, Sidak *post-hoc* test], with a *p*-values <0.05 considered significant. **p < 0.001, ^****^p < 0.0001.

Obtained compounds were evaluated using immortalised human adrenal cells expressing CYP17A1 and other steroidogenic enzymes. We first measured the ability of compounds to inhibit CYP17A1 hydroxylase and lyase reactions at 10 µM (Figure 3(A), Table S1). The more active compounds were then subjected to IC_50_ determination. From the initial screening, a set of SAR observations emerged. The most active compounds inhibiting the hydroxylase reaction were **5** and **11,** obtained in the same reaction. Those are the only two compounds containing indole moiety. Interestingly, compound **11,** the “di-cyano” product, was more potent than **5**. It was also more potent than abiraterone, used as a positive control in the assay. Similarly, when comparing the potency of another pair of “mono-cyano” and “di-cyano” compounds **1** and **10**, compound **10** was more potent. However, the compounds were less potent than **5** and **11**. This suggests that indole (in **5** and **11**) is a preferred substituent compared to the benzotriazole fragment (in **1** and **10**). Other compounds were significantly less active displaying <50% of enzyme inhibition at 10 µM concentration. Among those compounds some structural similarity can be identified between compound **6** and compounds **3** and **4**. They all have pyridyl and phenyl substituents around the central aromatic ring. Although in the case of the “mono-cyano” compounds **3** and **4**, it is meta-substituted pyridyl and in the case of the “di-cyano” **6** it is a para-substituted pyridyl group. In this case, we also found that **6** appeared to be more potent. Unfortunately, we were limited in comparison of the “mono-cyano” and “di-cyano” pairs due to the synthetic feasibility. However, even with these few examples, there is a trend to favour compounds with two cyano groups and a benzene core instead of pyridine.

Next, we were interested in measuring the ability of compounds to inhibit the CYP17A1 lyase reaction. Compounds which selectively inhibit the CYP17A1 lyase reaction over hydroxylase activity are considered to be burdened with fewer side effects^41^. Unfortunately, none of the compounds displayed a marked preference for lyase inhibition. However, it should be noted that compounds **1**, **5**, **10**, and **11** were among the most potent. During detailed IC_50_ determination compounds **11**, **1**, and **5** showed IC_50_ = 4, 10, and 25 nM, respectively (Figure 3(B)).

Four additional analogs (**12–15**), designed to validate compound **11** geometry, were tested confirming a preferred “bent” geometry, i.e. with a meta-substituted benzene core. Compounds **12** and **14** demonstrated greater potency than “straight” compounds **13** and **15**, which have a para-substituted benzene core, in both CYP17A1 hydroxylase and lyase inhibition assays (Figure 3(A), Table S1). Interestingly, the most potent compound inhibiting CYP17A1 lyase activity was **14** (IC_50_ = 26 nM) (Figure 3(B)). This finding validated the overall shape of the scaffold to which different substituents can be attached in prospective analogs.

### Steroid profiling

**Figure 4.**
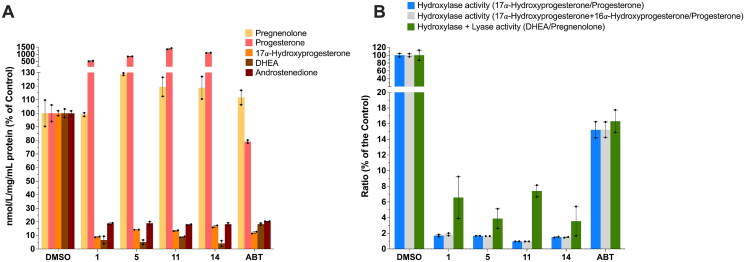
Steroid profile for selected compounds (**1**, **5**, **11**, **14**) and abiraterone (ABT) compared to DMSO control. (A) shows the changes in androgen-related steroid metabolites in response to treatment with DMSO, compounds **1**, **5**, **11**, **14** at 10 µM, and ABT. Each bar represents the average ± *SD* metabolite level for each treatment as a % of the control. (B) Shows the percentage activity of CYP17A1’s 17α-hydroxylase and combined with 17,20-lyase function, calculated as ratios, normalised to DMSO-treated cells (100%).

The effects of the selected compounds (**1**, **5**, **11**, **14**) and abiraterone (ABT) on CYP17A1 enzyme activities were further investigated using human adrenal NCI-H295R cells, through measuring steroid profiles by liquid chromatography-mass spectrometry (LC-MS), as previously published^42^^,^^43^. The data revealed that these compounds significantly altered the levels of key steroids, including 17α-hydroxyprogesterone, androstenedione, and DHEA, compared to the DMSO control, demonstrating their impact on steroidogenic pathways (Figure 4(A), Table S2).

For 17α-hydroxyprogesterone, the selected compounds demonstrated a high inhibitory activity, reducing its levels 7-fold on average. This activity was similar or higher for compound **1** than ABT. As expected, and due to the inhibition of CYP17A1, pregnenolone and progesterone levels increased in the presence of the selected compounds and ABT. The levels of 11-deoxycortisol were also reduced by the selected compounds with compound **14** showing the most substantial reduction from 26.59 to 3.75 nmol/L (Figure S1). This decrease could be due to the decreased substrate availability for 11-deoxycortisol biosynthesis. Additional investigations into cortisol biosynthesis could however not be completed, as cortisol was not detected within the timeframe of the experiment. Similarly, corticosterone, aldosterone, cortisone, DHEA-S, and dihydrotestosterone were not detected within the experimental timeframe.

Interestingly, 11-deoxycorticosterone levels did not decrease but increased in the presence of compound **1** (from 28.16 to 41.58 nmol/L), suggesting conversion of accumulated progesterone (due to CYP17A1 inhibition) in the presence of this compound. Conversely, similarly to ABT, compounds **5**, **11**, and **14** markedly decreased 11-deoxycorticosterone levels, suggesting inhibition of enzymes responsible for its biosynthesis (CYP21A2).

Androstenedione levels were reduced by all the selected compounds (5-fold), particularly compound **5**, which lowered androstenedione from 7.58 to 1.41 nmol/L. This decrease suggests inhibition of CYP17A1 lyase reaction, which is required for DHEA and subsequently androstenedione biosynthesis. While ABT also showed a reduction, the effect was not as strong as that of compounds **5**, **11**, and **14**, emphasising the improved potency of these new compounds. Testosterone levels remained relatively stable across all treatments, with only slight variations (notably, testosterone was detected at low levels).

We were most interested in the androgen precursor, DHEA, and the effect of the compounds on its biosynthesis, as DHEA is the direct result of the sequential catalytic activities of CYP17A1. The most significant reduction was observed in DHEA levels. Compound **11** lowered DHEA from 15.12 to 1.36 nmol/L (11-fold), reflecting a potent inhibition of 17α-hydroxylase and 17,20-lyase activities of CYP17A1. ABT also reduced DHEA, but the effect was less pronounced compared to all the selected compounds, underscoring again the higher potency of the compounds in inhibiting DHEA biosynthesis.

To provide deeper insights into CYP17A1 inhibition, the enzyme activity was calculated using ratios of steroid products to substrates, focusing on dual activities of CYP17A1 (Figure 4(B)). For 17α-Hydroxylase activity, compound **11** exhibited the most potent inhibition, reducing hydroxylase activity to 1%, followed by compounds **5** and **14**, which lowered it to 1.7 and 1.5%, respectively. Compound **1** showed a slightly higher activity at 1.8%, while ABT only reduced the hydroxylase activity to 15.2%. For combined 17α-hydroxylase and 17,20-lyase activity, compound **11** also demonstrated potent inhibition, reducing the CYP17A1 activity to 7.4%, followed by compounds **14** (3.5%) and **5** (3.9%). Compound **1** exhibited a slightly higher CYP17A1 activity of 6.6%, while ABT maintained ∼16.3% of the enzyme’s activity. It should be mentioned that 17α-hydroxypregnenolone could unfortunately not be quantified in this experiment to serve as the direct substrate for calculating the 17,20-lyase activity separate from the hydroxylase activity.

These enzyme activity determinations highlight the potent inhibitory effects of compounds **1**, **5**, **11**, and **14** on CYP17A1, particularly compound **11**, which showed high inhibitory activity across both enzymatic functions, indicating a potential high degree of selectivity in targeting CYP17A1.

### Impact on prostate cancer cells

Since our research has been focused on discovery of novel prostate cancer CYP17A1 modalities we wanted to check the influence of the obtained compounds on prostate cancer cell lines. We used LNCaP and VCaP cell lines as a model of androgen sensitive PCa. Cell viability was assessed at two time points, 24 and 48 h, to capture both immediate and delayed effects of the treatments. The VCaP and LNCaP cell lines depend on androgen signalling for proliferation, with VCaP cells derived from a vertebral metastasis and LNCaP cells originating from a lymph node metastasis, making them highly relevant models for studying androgen receptor (AR)-driven prostate cancer progression and the therapeutic effects of androgen-targeting treatments^44^^,^^45^.

**Figure 5.**
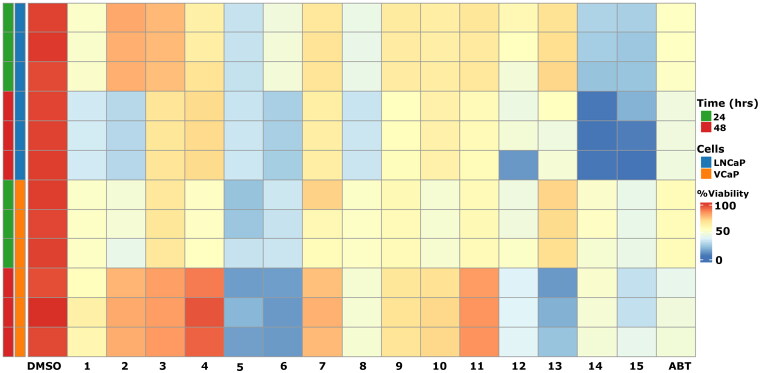
Cytotoxicity assessment of various compounds in VCaP and LNCaP prostate cancer cell lines using the resazurin assay. The heatmap displays cell viability following treatment with 10 µM of each compound at 24 and 48 h. DMSO served as a negative control, while abiraterone (ABT) was used as a positive control. Results from three independent experiments are presented for each compound.

We found that the viability of androgen-sensitive prostate cancer cells (VCaP and LNCaP) significantly decreased in response to most compounds (Figure 5). In the VCaP cytotoxicity assay, several compounds reduced cell viability to below 50% after 24 h, with compounds **5** and **6** showing the most pronounced effects. After 48 h, these effects became even more pronounced, indicating that prolonged inhibition of androgen synthesis through sustained CYP17A1 blockade leads to further reduction in cell survival. This highlights the efficacy of these CYP17A1-targeted compounds in impairing androgen-dependent tumour growth over time. Similarly, in the LNCaP cytotoxicity assay, compounds **5** and **6** caused a marked reduction in viability, consistent with the VCaP results. Interestingly, while compound **11** showed the most potent inhibition of CYP17A1 enzyme activity, as evidenced by LC-MS steroid profile analysis and other assays, it did not exhibit high cytotoxic effects on VCaP or LNCaP cells. This suggests that although compound **11** is effective in targeting androgen biosynthesis, its lack of cytotoxicity in these cell lines indicates a more selective mechanism of action. This highlights the potential for compound **11** to inhibit androgen production without directly compromising cell viability, offering a different therapeutic profile compared to other compounds that exhibit both enzyme inhibition and cytotoxicity. On the other hand, compounds **14** and **15** displayed strong effect in LNCaP cells after 48 h. This effect was much less pronounced in VCaP cells. Given that compound **14** is also a potent inhibitor, with a preference towards lyase reaction, this could indicate increased sensitivity of LNCaP cells to lyase inhibition. It should be noted that VCaP cells have longer doubling time than LNCaP cells and in relation to compound **14** they could be less affected after 48 h. Abiraterone, the positive control, showed a notable reduction in cell viability in both VCaP and LNCaP cells. The differential responsiveness observed between the compounds and abiraterone highlights the potential of the compounds, particularly compound **5**, to exert broad-spectrum cytotoxic effects by effectively disrupting androgen biosynthesis and impairing androgen receptor signalling in PCa cells.

Importantly, none of the tested compounds exhibited significant cytotoxic effects on the normal prostate cell line RWPE-1 (Figure S2). Only compound **12** showed a mild reduction in cell viability, decreasing it to 85% of the control after 48 h. Our compounds showed low to moderate variability in increased cellular metabolic activity suggesting consistent response to the treatment. Notably, abiraterone demonstrated higher toxicity, reducing cell viability to 84% after 24 h and further to 66% after 48 h. These effects of abiraterone, which inhibit cell proliferation through the suppression of the Wnt/β-catenin signalling pathway, have been previously documented in both *in vitro* and *in vivo* studies^46^^,^^47^.

### Impact on cell migration

**Figure 6.**
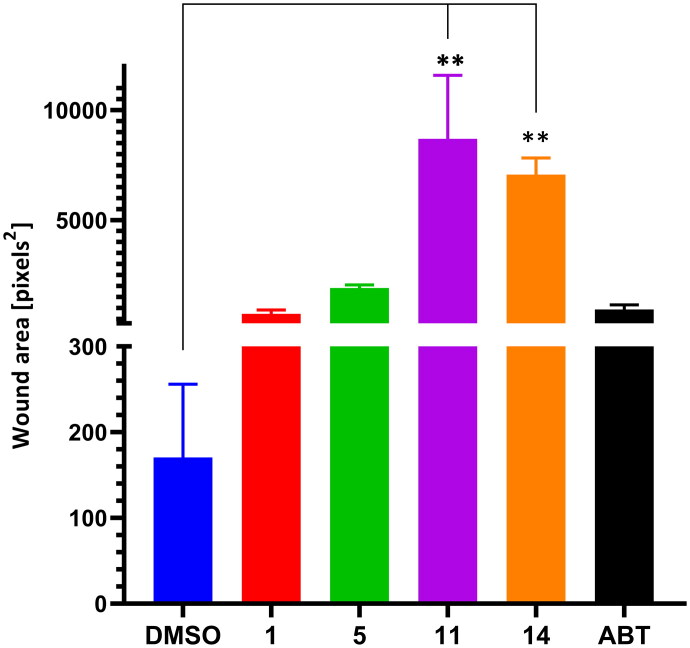
Impact of selected compounds on cell migration in the wound healing assay showing quantitative analysis of wound closure. Data are expressed as the pixels from the image of wound area remaining open after 24 h, with DMSO as the control. Treatment with **11** and **14** significantly inhibited cell migration, as evidenced by a larger remaining wound area compared to the control. The data indicates the relative efficacy of each compound in affecting cell migration. Values are presented as mean ± standard deviation from three independent experiments. Statistical significance was determined using One-way ANNOVA, [Dunnet’s *post-hoc* test], with a *p*-values <0.05 considered significant. **p < 0.01.

The effect of the selected compounds on the migratory ability of prostate cancer cells was examined by wound healing assay using DU-145 prostate cancer cells due to their distinctive dense growth pattern which allows creating a wound and monitoring it by imaging analysis. The representative control (DMSO) demonstrates that the scratch wound was almost completely closed within 24 h (Figure S3). To quantify the effects of the compounds on cell migration, we measured the area of the open wound (Figure 6, Table S3). Our results revealed that treatment with **11** and **14** resulted in significant inhibition of cell migration after 24 h, indicating their potent effects on reducing cell movement and potentially impairing wound healing. In contrast, **1** and **5** exhibited less pronounced effect comparable to that exerted by abiraterone. These findings suggest that **11** and **14** may effectively hinder cell migration, which could have implications for their role in preventing the spread of PCa cells.

### Molecular modelling

To investigate the effects of the substitution pattern on the central benzene ring (meta- *vs.* para-substituted) and the pyridine ring (meta- *vs.* para-substituted) compounds **12**–**15** were docked to CYP17A1 protein (PDB 3SWZ). Only the two compounds with a meta-substituted pyridine moiety, **12** and **13** were able to adopt a binding mode with direct coordination between the pyridine and the haem group (N-Fe = 2.44 and 2.50 Å for **12** and **13**, respectively) (Table 1). For the two compounds with a para-substituted pyridine moiety, **14** and **15**, docking did not identify any poses with an N-Fe distance indicating a coordination of the ligand to the haem group.

**Table 1.** GOLD docking data.

Compound^a^	GOLD score^b^	Δ*G* binding^c^	Fe-N distance (Å)	Fe facing entity/comment
**1**	55.39	−59.76	2.40	Benzotriazole N2
**1** (N)	39.12	−48.89	2.51	Para-substituted pyridine N
**1** (N)	38.49	−52.00	2.26	Para-substituted pyridine N
**2**	39.80	−40.08	5.08	Central pyridine N
**3**	44.46	−45.50	2.43	Meta-substituted pyridine N
**4**	40.04	−40.97	4.65	Central pyridine N
**5**	40.25	−41.06	No Fe-N contact	Indole
**5** (N)	39.43	−47.04	2.56	Para-substituted pyridine N
**6**	35.79	−36.18	5.09	Cyano N
**7**	42.19	−45.93	2.54	Meta-substituted pyridine N
**8**	36.52	−36.69	5.24	Cyano N
**9**	42.19	−45.93	2.54	Meta-substituted pyridine N
**10**	52.34	−62.58	2.42	Benzotriazole N2
**10** (N)	36.52	−49.71	2.54	Para-substituted pyridine N
**11**	40.57	−41.66	5.21	Cyano N
**11** (N)	37.41	−48.83	2.56	Para-substituted pyridine N
**11** (N)	33.91	−48.09	2.50	Para-substituted pyridine N
**12**	47.87	−53.97	2.44	Meta-substituted pyridine N
**13**	48.86	−50.88	2.50	Meta-substituted pyridine N
**14**	46.93	−47.60	No Fe-N contact	Para-substituted pyridine N
**14** (N)	46.35	−49.68	2.50	Para-substituted pyridine N
**15**	44.12	−44.62	4.28	Indole NH
**15**	42.76	−43.61	3.42	Para-substituted pyridine N/poor Fe-N geometry
**15** (N)	43.94	−49.62	2.59	Para-substituted pyridine N

^a^ The N in bracket refers to a GOLD docking with the pyridine N-Fe distance constrained.

^b^ The GOLD score is derived from the ΔG value by adding clash penalty and internal torsion terms.

^c^ The Δ*G* is an estimate of the free energy of binding. See GOLD User Guide (www.ccdc.cam.ac.uk/media/Documentation/GOLD_User_Guide_2020_1.pdf).

Docking of the following group of compounds, **2**–**4** and **6**–**9** showed again that the compounds with a meta-substituted pyridine moiety **3**, **7**, and **9** easily could fit into the CYP17A1 binding site with N-Fe distances on 2.43, 2.54, and 2.54 Å, respectively (Table 1). Although compound **4** also contains a meta-substituted pyridine, we did not observe any poses with the pyridine facing the haem group. For compounds **2**, **6**, and **8** no ligand group coordinated to the Fe atom in the haem group. This group of compounds seems to suggest a preference for a meta-substituted pyridine compared to a para-substituted pyridine for establishing a proper ligand-haem coordination as exemplified by compound **9**, which contains both a meta- and a para-substituted pyridine moiety.

The third group of compounds comprise the cyano-containing potent inhibitors **1**, **5**, **10**, and **11**. Compounds **1** and **10** contain two potential haem-coordinating groups, and that both the pyridine and benzotriazole may coordinate to the haem group is evident from the X-ray structures of CYP17A1 with abiraterone (3RUK) and galeterone (3SWZ), respectively. The binding mode of compound **1** to CYP17A1 has already been studied in the original paper by Bonomo et al.^18^, and DFT calculations on various heteroaromatic systems and experimental binding studies of analogues of **1** showed that the pyridine moiety was superior to the benzotriazole moiety in making favourable interactions with a haem group.

**Figure 7.**
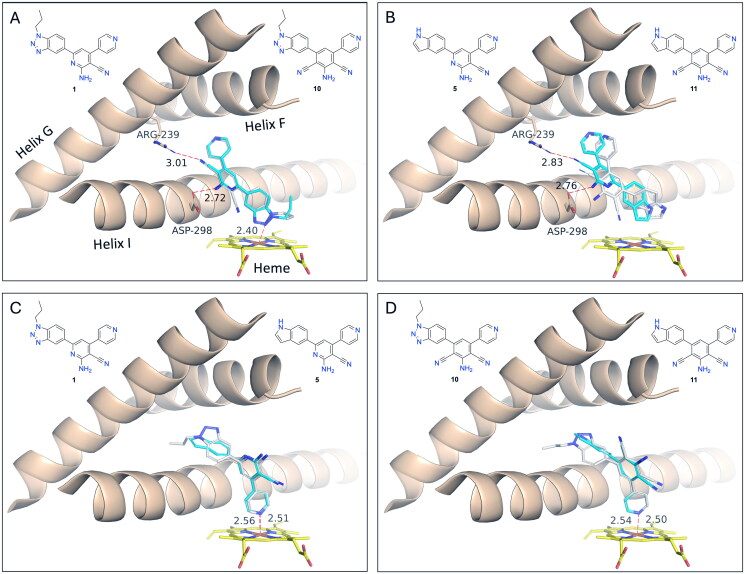
GOLD predicted binding modes to CYP17A1. (A) **1** (C-atoms cyan) and **10** (C-atoms white) from GOLD docking without constraints. Only contacts between **1** and CYP17A1 are shown. (B) **5** (C-atoms cyan) and **11** (C-atoms white) from GOLD docking without constraints. Only contacts between **5** and CYP17A1 are shown. (C) **1** (C-atoms cyan) and **5** (C-atoms white) from GOLD docking with constraints. Only contacts between **1** and CYP17A1 are shown. (D) **10** (C-atoms cyan) and **11** (C-atoms white) from GOLD docking with constraints. Only contacts between **10** and CYP17A1 are shown.

Docking of **1** as well as **10** only finds binding modes with the benzotriazole N2 atom coordinating to the Fe atom (Figure 7(A)). Surprisingly, compounds **5** and **11** bind analogously with the indole moiety placed as the benzotriazole moiety in **1** and **10**, although it does not contain any heteroatom with a lone-pair available for interaction with the electrophilic Fe atom (Figure 7(B)). The only other specific interactions are hydrogen bonds with Arg 239 and Asp298 in **1**, **5**, and **10**, whereas the corresponding distances in **11** are too long to be classified as hydrogen bonds. We consider the binding mode of these compounds with the indole/benzotriazole moieties facing the haem group primarily to be determined by the overall shape of the compounds rather than by specific interactions.

To investigate, if compounds **1**, **5**, **10**, and **11** could adopt binding modes with the pyridine N coordinating to Fe, we subjected these compounds to a constrained docking. For all four compounds we obtained docking poses with pyridine N-Fe distances of 2.53 ± 0.03 Å (Figures 7(C,D)).

The stability of the binding mode obtained by docking was investigated by MD simulations of **1** and **10** with the benzotriazole coordinating to the haem group (Figure S4). Compound **1** is immediately shifted away from the haem group and is positioned with an N-Fe distance on ∼7.5 Å where it remains stable for the remaining simulation time. Compound **10** is slowly drifting from a conformation with the N-Fe distance increasing from ∼3 Å towards ∼5 Å. Thus, we must conclude that for both **1** and **10**, conformations with the benzotriazole facing the haem group do not represent likely binding modes.

The poses obtained by constrained docking may not necessarily represent favourable binding modes. Thus, we have subjected these poses of compounds **1**, **5**, **10**, and **11** to short MD simulations. For all four compounds, we observed some initial change in binding mode from the poses from the constrained docking, typically already from frame 1 (after equilibration) to frame 2 (after 1 ns simulation), but subsequently the compounds maintain a stable binding mode.

For compound **1** the coordination to the haem group is constant during the MD simulation (N-Fe distance = 2.32 ± 0.09 and 2.41 ± 0.20 Å). A hydrogen bond from the amino group to Leu370 (75% of the frames), water-mediated hydrogen-bonds from Thr306 and Gly301 to benzotriazole N3 (86 and 92%, respectively) as well as a π-π contact between Phe114 and the central pyridine ring (83%) are all present in more than 75% of the simulation time. The torsional angle between the two pyridine groups changes within the first few frames from ∼125 to ∼45° for the remaining part of the simulation. Compound **10** maintains an N-Fe distance of 2.37 ± 0.11 Å during the MD simulation. A hydrogen bond from the amino group to Leu370 is present in 51% of the frames, but generally, we observe fewer specific interactions for compound **10** compared to **1**.

For compounds **5** and **11**, MD simulations without any restrictions revealed that the conformations from the constrained docking remain stable after the initial shift from the poses from the constrained docking. The binding of compound **5** is primarily fixed by the coordination to the haem group and a hydrogen bond from the indole NH group to the side-chain oxygen atom in Asn202 present in 83 and 94% of the simulation, respectively. In **5** the torsional angle between the two pyridine groups remains similar to that observed from the docking pose/initial value (∼125°). The binding of compound **11** closely resembles the binding of compound **5** with the pyridine N atom coordinating to the Fe in the haem group (95% of the frames) and a hydrogen bond to Asn202 (100% of the frames). An especially interesting feature is observed in the MD simulations of **5** and **11**. The N-Fe distance increases beyond 5 Å for 16 and 5 frames, respectively, and the direct coordination is lost, but that the N-Fe coordination is re-established for the remaining part of the simulation, indicating that this binding mode is more stable. Finally, it is worth mentioning that the indole containing compounds **5** and **11** are characterised by a smaller RMSD relative to the protein (RMSD = 1.1–1.8 Å) than the corresponding benzotriazole analogues **1** and **10** (RMSD = 3.1–4.2 Å) indicating a tighter fit between ligand and protein in agreement with the gain in affinity by replacing the benzotriazole moiety with an indole moiety.

**Figure 8.**
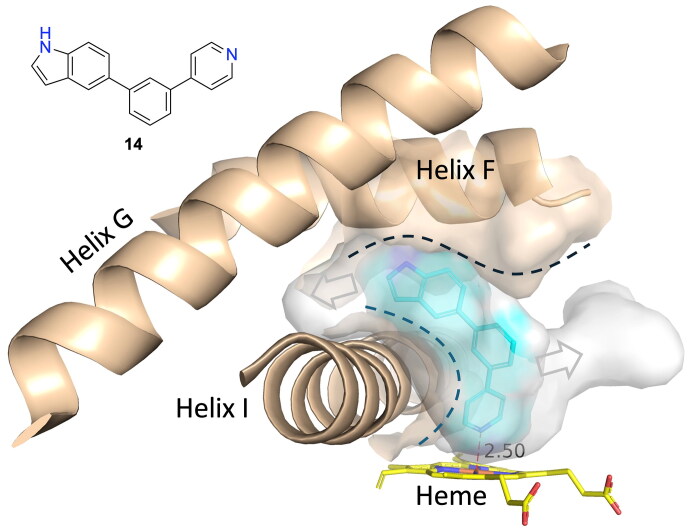
Binding of **14** to CYP17A1. The surface of Asn202, Ile205, Ile206, and Leu209 on Helix F and Gly301, Ala302, and Thr306 on Helix I are the only residues in direct contact with compound **14**. The dotted lines illustrate the shape of the contact between compound **14** and the surfaces of Asn202, Ile205, Ile206, and Leu209 in Helix F and Gly301, Ala302, and Thr306 in Helix I. The arrows indicate space not occupied by **14** and potential space for substituents.

Compound **14** is interesting from the perspective it can be considered as the parent compound for **5** and **11**. MD simulations show that the binding conformation with the pyridine ring facing the haem group is stable. Also, it shows that the compounds do not make any polar/hydrogen-bonding contacts with the protein. Docking of compound **14** (Figure 8) showed it curled around helix I, filling the cavity of the active site and thus confirming its geometry to be the most optimal. Compounds **13** and **15** are linear while compound **12** has pyridine with a nitrogen atom in the para- position which makes them less capable of accommodating the optimal position in the active site. Those observations suggest that compound **14** can serve as a template for further design efforts incorporating alterations, such as an addition of functional groups compatible with neighbouring amino acids or swapping the central aromatic core for bioisostere richer in sp^3^ carbon atoms^48^^,^^49^.

In this study, docking without constraints didn’t identify all likely binding modes. By constrained docking additional binding modes were generated, and MD simulations were required to identify stable and relevant binding modes.

## Conclusions

Cytochrome P450 enzyme CYP17A1 is an established target for PCa treatment. Multiple CYP17A1 inhibitors have been in development for many years. However, only one compound, abiraterone, has been approved for clinical use. Abiraterone has a steroid core structure and as such cause several side effects. In the present work, we focus on non-steroidal compounds targeting CYP17A1 to obtain inhibitors with improved properties.

Starting with structure **1** we designed analogs to determine a structure-activity relationship for this compound series. During their synthesis, we encountered unexpected reaction products which upon isolation and structure elucidation were tested for biological activity. Among these, compound **11** markedly influenced the activity of CYP17A1 in human adrenal cells, achieving an IC_50_ value in the nanomolar range (IC_50_ = 4 nM). In a subsequent LC-MS analysis of DHEA level, an important androgen precursor, compound **11** was found more potent than the benchmark compound abiraterone. Antiproliferative assays on androgen sensitive LNCaP and VCaP did not show significant activity of compound **11**. However, a structurally similar compound **14** gave potent reduction of LNCaP cell viability. This compound was synthesised as an analog of **11** to confirm the preferred scaffold geometry and devise a template upon which further modifications can be incorporated. This compound also proved to be potent in the CYP17A1 assays, although its potency was one order of magnitude lower (IC_50_ = 26 nM) than that of compound **11**. Importantly, **14** showed a preference to inhibit the CYP17A1 catalysed lyase reaction over the hydroxylase reaction, a preferred pharmacological profile of CYP17A1 inhibitors. Additionally, both **11** and **14** demonstrated substantial impact on cell migration in the wound healing assay using metastatic DU-145 cells, indicating potential role in preventing metastasis of PCa cells. Computational chemistry studies combining docking and MD calculations showed that compounds **1** and **10** can adopt two different binding modes with either the pyridine or the benzotriazole moiety coordinating to the Fe atom in the haem group. The pyridine is the most likely Fe-coordinating group in agreement with the previous study on compound **1**^18^. Our analysis also showed that all the most potent compounds, **1**, **5**, **11**, and **14** may adopt stable binding modes to CYP17A1 with the pyridine N atom coordinating to the Fe atom in the haem group. Our MD simulations also revealed that the indole containing compounds **5** and **11** display less movement in the binding site compared to the benzotriazole containing analogues **1** and **10**. Generally, the compounds seem primarily to bind by favourable non-bonded interactions.

In summary, we have transformed the hit compound **1** into two promising lead compounds, compound **11** was found more active than abiraterone, and compound **14** was found more selective towards the CYP17A1 lyase reaction.

## Supplementary Material

supp_info_JEIMC_ Clean.docx

## Data Availability

The data that support the findings of this study are openly available in RepOD Repository for Open Data https://doi.org/10.18150/9PQHUY.
