# Supplementary material for: Pyridine indole hybrids as novel potent CYP17A1 inhibitors
Source: J Enzyme Inhib Med Chem. 2025 Feb 14;40(1):2463014. doi: 10.1080/14756366.2025.2463014 (PMC11834790; doi:10.1080/14756366.2025.2463014)

Serendipity Reveals Potent Inhibitors of CYP17A1

Tomasz M. Wróbel, Angelika Grudzińska, Jibira Yakubu, Therina du Toit, Katyayani Sharma, Jeremiah C. Harrington, Natalia Dycha, Fredrik Björkling, Flemming Steen Jørgensen and Amit V. Pandey

**Supplementary Information**

Table of Contents

[Supplementary figures 2](#_Toc183163144)

[Fig. S1 Hormones levels measured after exposure to the tested compounds. 2](#_Toc183163145)

[Fig. S2 Effect of the tested compounds on normal prostate cells RWPE-1. 3](#_Toc183163146)

[Fig. S3 Wound healing assay images. 4](#_Toc183163147)

[Fig. S4 Fe-N distances from MD simulations. 5](#_Toc183163148)

[Supplementary tables 6](#_Toc183163149)

[Table S1. The CYP17A1 hydroxylase and lyase activity of the compounds 6](#_Toc183163150)

[Table S2. LC-MS steroid profile of the compounds 7](#_Toc183163151)

[Table S3. Wound closure expressed as pixel square 8](#_Toc183163152)

[NMR and LC-MS data 9](#_Toc183163153)

[Compound 1 9](#_Toc183163154)

[Compound 2 11](#_Toc183163155)

[Compound 3 13](#_Toc183163156)

[Compound 4 15](#_Toc183163157)

[Compound 5 17](#_Toc183163158)

[Compound 6 19](#_Toc183163159)

[Compound 7 21](#_Toc183163160)

[Compound 8 23](#_Toc183163161)

[Compound 9 25](#_Toc183163162)

[Compound 10 27](#_Toc183163163)

[Compound 11 29](#_Toc183163164)

[Compound 12 31](#_Toc183163165)

[Compound 13 33](#_Toc183163166)

[Compound 14 35](#_Toc183163167)

[Compound 15 37](#_Toc183163168)

# Supplementary figures

## Fig. S1 Hormones levels measured after exposure to the tested compounds.


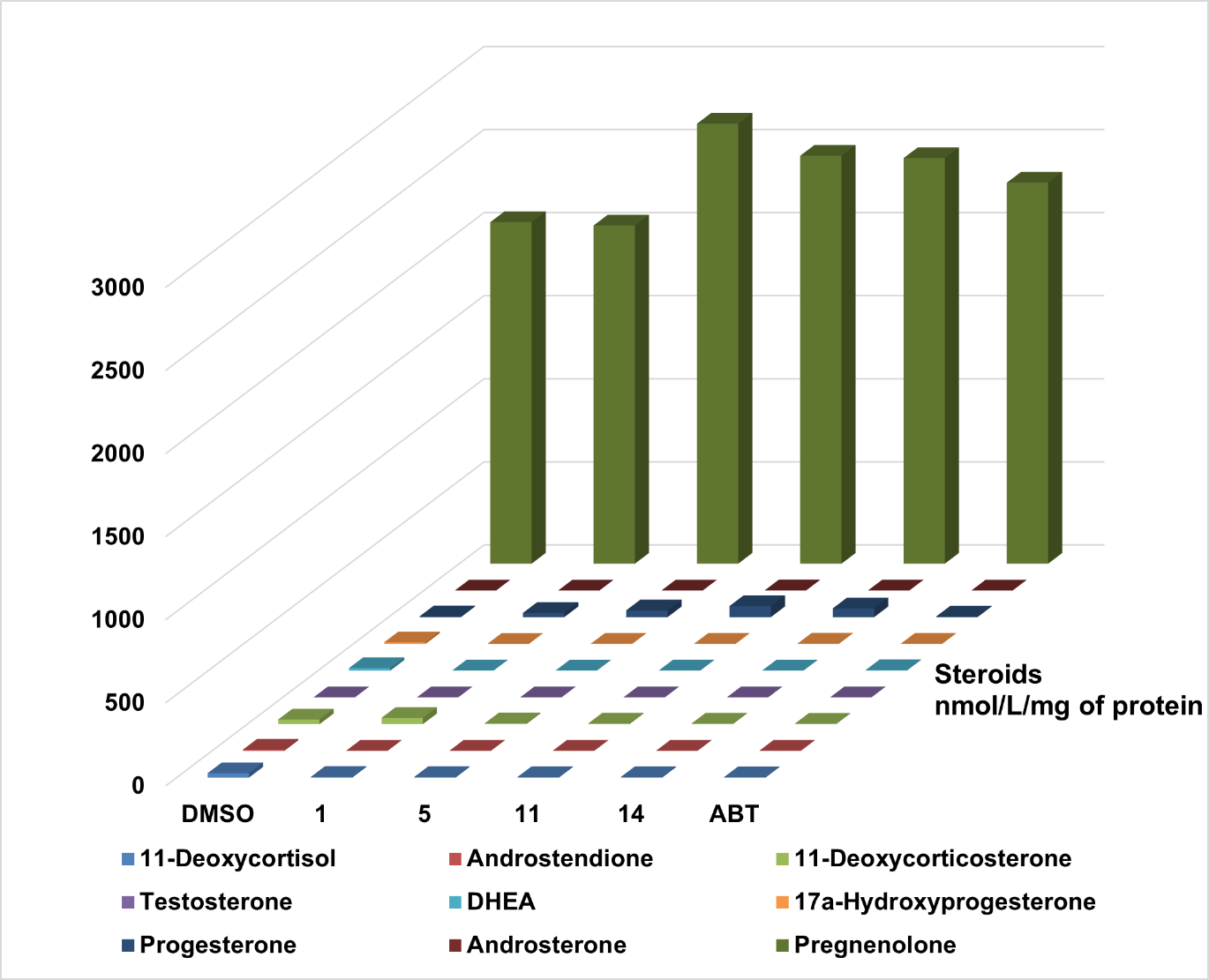


## Fig. S2 Effect of the tested compounds on normal prostate cells RWPE-1.

Cell viability of RWPE-1 cells following treatment with compounds 1-15, Abiraterone (ABT), and DMSO (dimethyl sulfoxide) as the control was assessed by the resazurin assay. The resazurin assay measures cellular metabolic activity, which is indicative of cell viability. The data are presented as the mean ± standard deviation (SD) of three independent experiments (n = 3). Bars represent the variability of cell viability across replicates, with higher values indicating greater cell viability in response to the treatments.


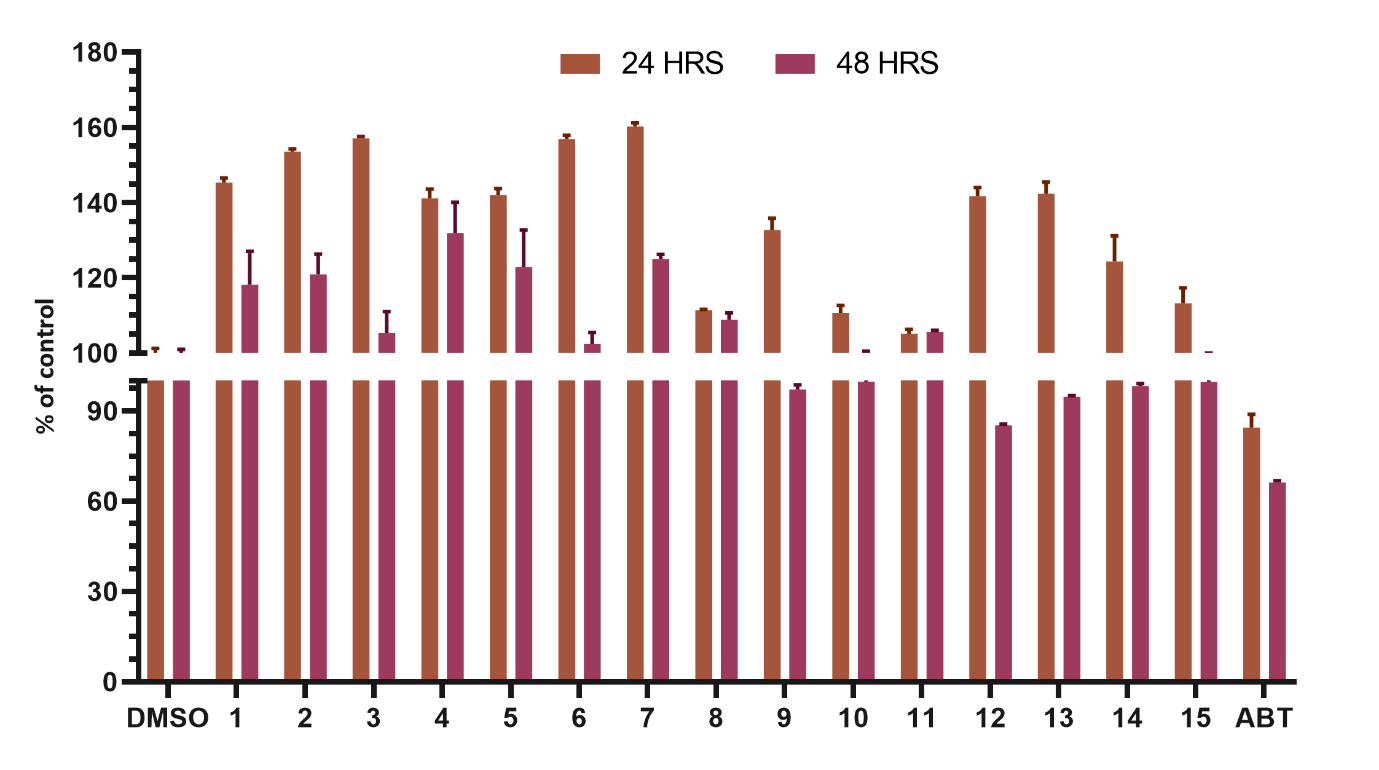


## Fig. S3 Wound healing assay images.


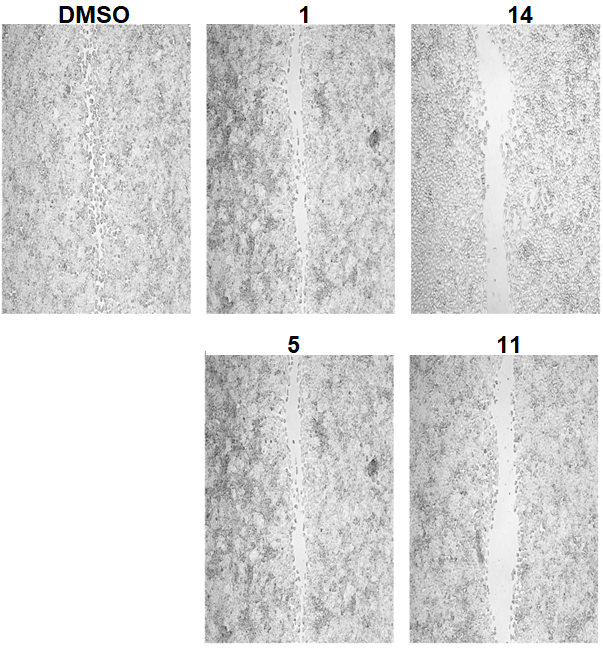


## Fig. S4 Fe-N distances from MD simulations.


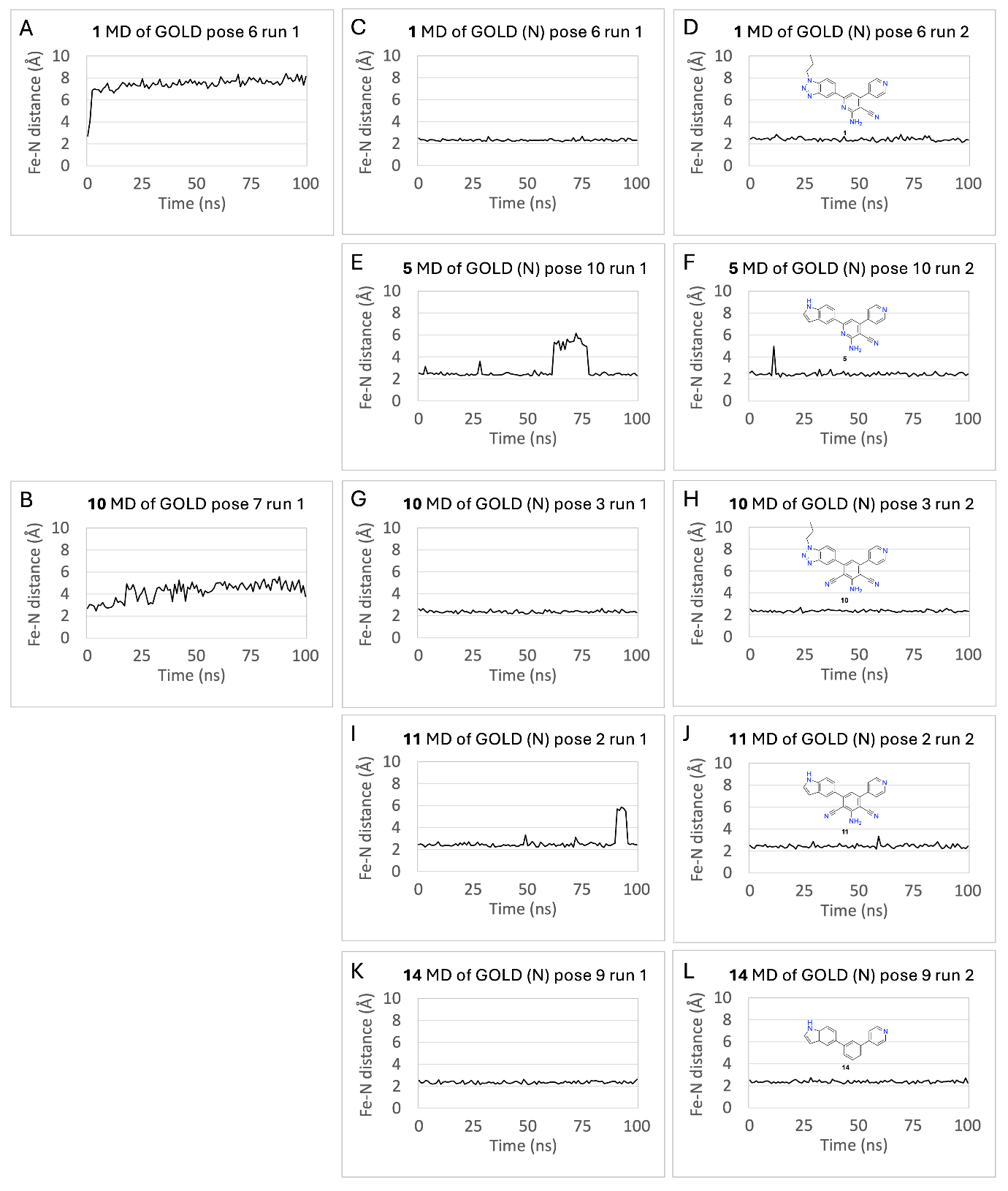


# Supplementary tables

## Table S1. The CYP17A1 hydroxylase and lyase activity of the compounds

|  | Hydroxylase | | | Lyase | | |
| --- | --- | --- | --- | --- | --- | --- |
|  | Mean | SD | Adjusted P-value | Mean | SD | Adjusted P-value |
| DMSO | 100 | 3.69 | NA | 100 | 0.68 | NA |
| 1 | 45 | 3.86 | <0,0001 | 46.29 | 2.54 | <0,0001 |
| 2 | 67 | 12.64 | <0,0001 | 93.66 | 13.7 | >0,9999 |
| 3 | 84 | 2.88 | 0.3844 | 94.74 | 1.35 | >0,9999 |
| 4 | 71 | 15.52 | <0,0001 | 68.54 | 3.55 | <0,0001 |
| 5 | 20 | 0.84 | <0,0001 | 39.71 | 5.75 | <0,0001 |
| 6 | 53 | 3.62 | <0,0001 | 57.42 | 2.71 | <0,0001 |
| 7 | 96 | 4.74 | >0,9999 | 112.56 | 13.03 | 0.9411 |
| 8 | 103 | 0.94 | >0,9999 | 84.81 | 8.29 | 0.5286 |
| 9 | 63 | 8.76 | <0,0001 | 66.75 | 1.01 | <0,0001 |
| 10 | 32 | 2.48 | <0,0001 | 58.25 | 6.6 | <0,0001 |
| 11 | 10 | 1.52 | <0,0001 | 49.16 | 2.54 | <0,0001 |
| 12 | 46 | 2.33 | <0,0001 | 48.71 | 4.3 | <0,0001 |
| 13 | 76 | 0.09 | 0.0033 | 93.46 | 0.39 | >0,9999 |
| 14 | 35 | 4.37 | <0,0001 | 23.85 | 5.86 | <0,0001 |
| 15 | 87 | 3.23 | 0.899 | 57.28 | 14.06 | <0,0001 |
| ABT | 14 | 4.46 | <0,0001 | 24.04 | 5.24 | <0,0001 |

## Table S2. LC-MS steroid profile of the compounds

|  | DMSO | | 1 | | 5 | | 11 | | 14 | | ABT | |
| --- | --- | --- | --- | --- | --- | --- | --- | --- | --- | --- | --- | --- |
| Steroids_nmol_per_L | Mean | SD | Mean | SD | Mean | SD | Mean | SD | Mean | SD | Mean | SD |
| 11-Deoxycortisol | 26.594 | 1.61786 | 5.22 | 0.041012 | 4.0415 | 0.159099 | 3.942 | 0.108894 | 3.7485 | 0.036062 | 3.585 | 0.043841 |
| Androstendione | 7.577 | 0.185262 | 1.564 | 0.059397 | 1.4095 | 0.119501 | 1.3725 | 0.037477 | 1.359 | 0.094752 | 1.5465 | 0.00495 |
| 11-Deoxycorticosterone | 28.1555 | 2.141826 | 41.577 | 0.168291 | 5.503 | 0.612354 | 3.8565 | 0.262337 | 3.518 | 0.015556 | 2.6135 | 0.037477 |
| Testosterone | 0.4785 | 0.03182 | 0.4455 | 0.014849 | 0.5215 | 0.047376 | 0.4775 | 0.070004 | 0.4105 | 0.010607 | 0.5005 | 0.002121 |
| DHEA | 15.119 | 0.663266 | 1.1015 | 0.649831 | 0.748 | 0.333754 | 1.3585 | 0.079903 | 0.6075 | 0.409415 | 2.775 | 0.156978 |
| 17a-Hydroxyprogesterone | 9.967 | 0.25173 | 0.977 | 0.043841 | 1.3865 | 0.009192 | 1.3575 | 0.047376 | 1.6245 | 0.098288 | 1.202 | 0.091924 |
| Progesterone | 4.814 | 0.41295 | 27.8235 | 1.692107 | 39.9815 | 0.381131 | 67.3265 | 2.833377 | 51.7765 | 0.415072 | 3.809 | 0.070711 |
| Androsterone | 0 | 0 | 0 | 0 | 0.009 | 0.012728 | 1.176 | 0.295571 | 0.61 | 0.321026 | 0 | 0 |
| Pregnenolone | 2043.56 | 280.4619 | 2228.179 | 39.87941 | 2578.994 | 23.5806 | 2465.505 | 204.4762 | 2377.463 | 233.9314 | 2279.668 | 154.2221 |
| Cortisone | 0 | 0 | 0 | 0 | 0 | 0 | 0 | 0 | 0 | 0 | 0 | 0 |
| DHEA-S | 0 | 0 | 0 | 0 | 0 | 0 | 0 | 0 | 0 | 0 | 0 | 0 |
| Dihydrotestosterone | 0 | 0 | 0 | 0 | 0 | 0 | 0 | 0 | 0 | 0 | 0 | 0 |

## Table S3. Wound closure expressed as pixel square

|  | Wound area (Pixels^2^) | | |
| --- | --- | --- | --- |
|  | Mean | SD | Adjusted P-value |
| DMSO | 170.5 | 85.56 | NA |
| 1 | 731.5 | 184.6 | 0.9843 |
| 5 | 1916 | 139.3 | 0.5288 |
| 11 | 8703 | 2886 | 0.0016 |
| 14 | 7074 | 748.8 | 0.0048 |
| ABT | 935 | 210.7 | 0.9478 |

# NMR and LC-MS data

## Compound 1

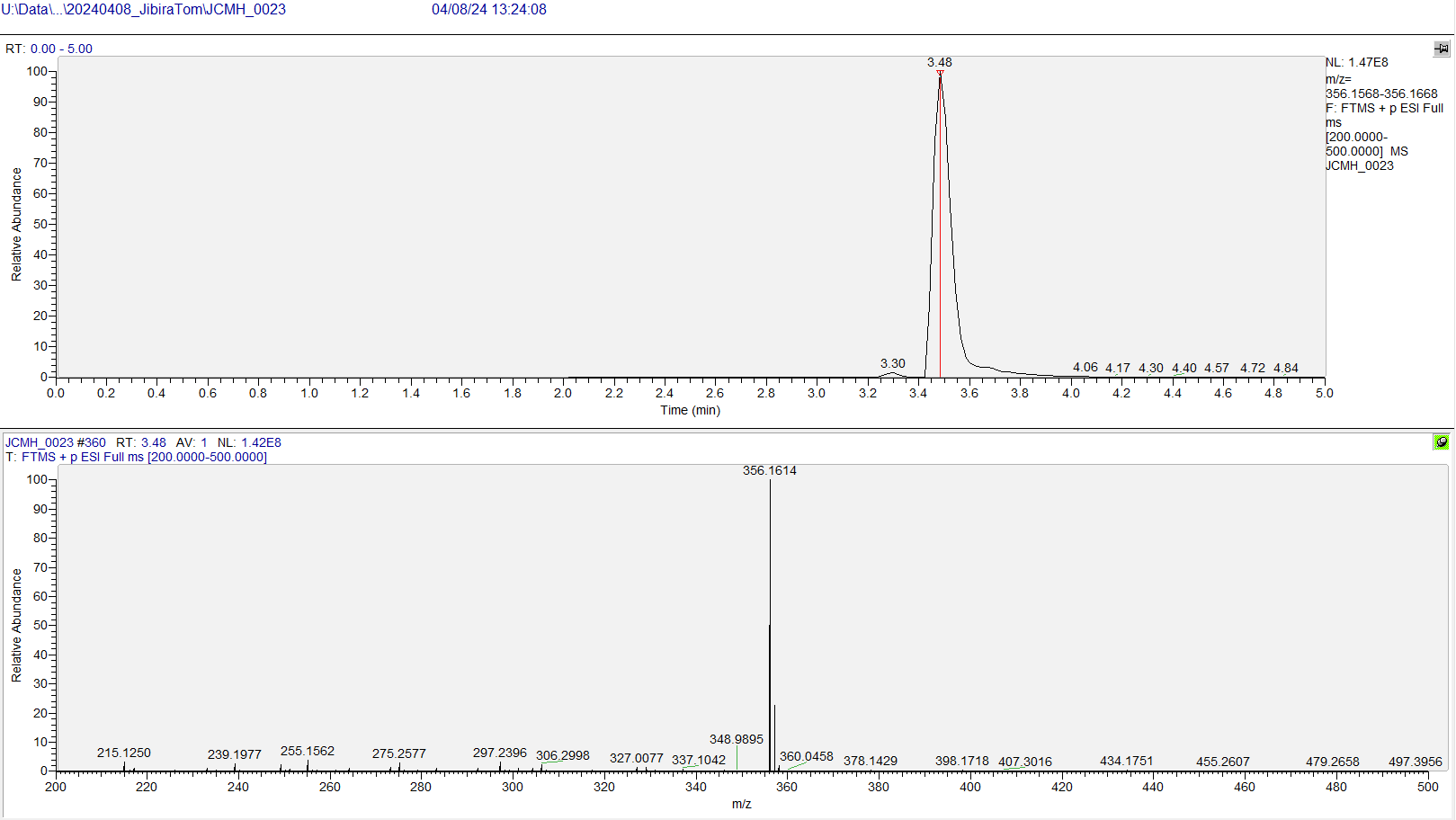


## Compound 2

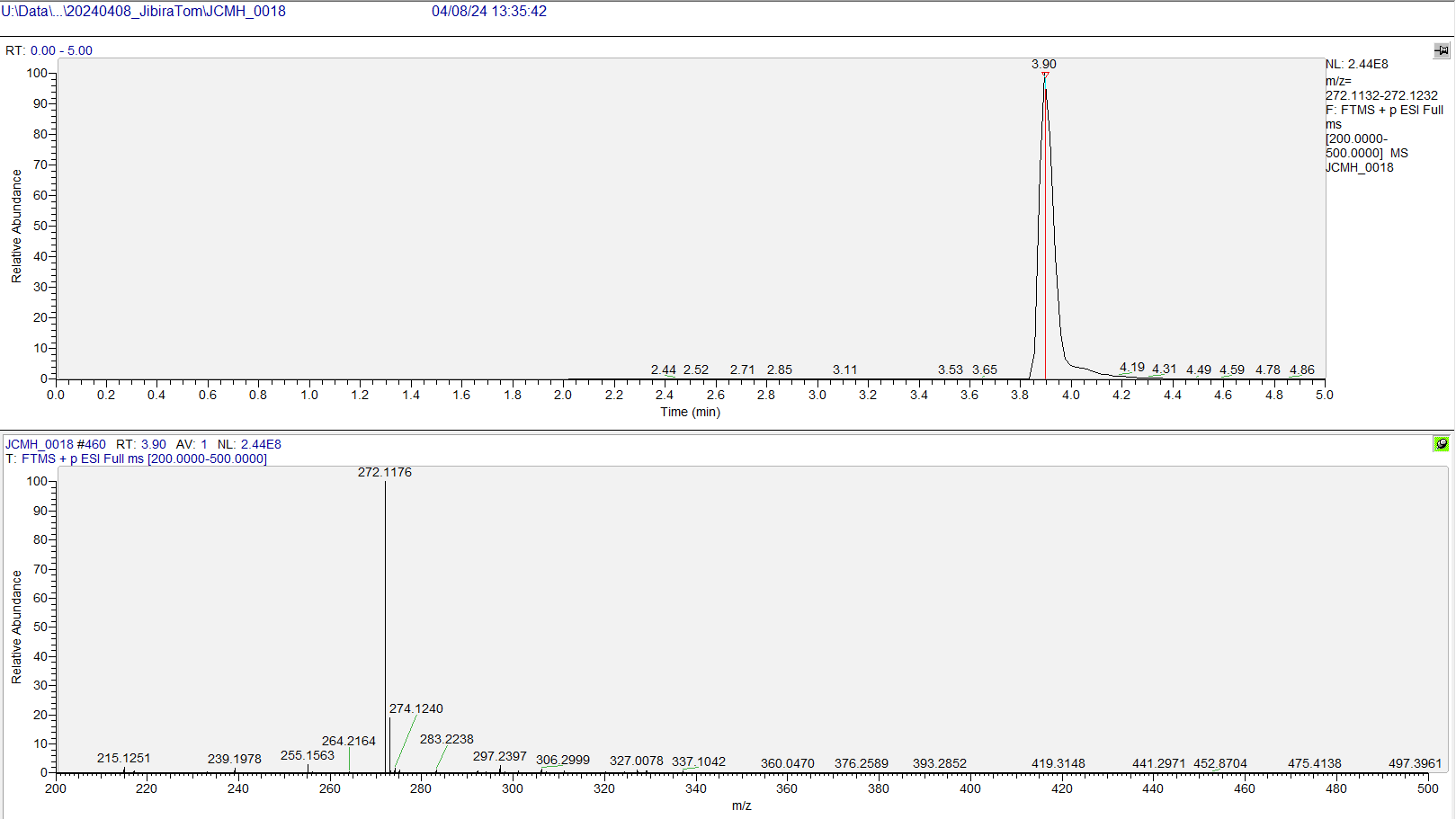


## Compound 3

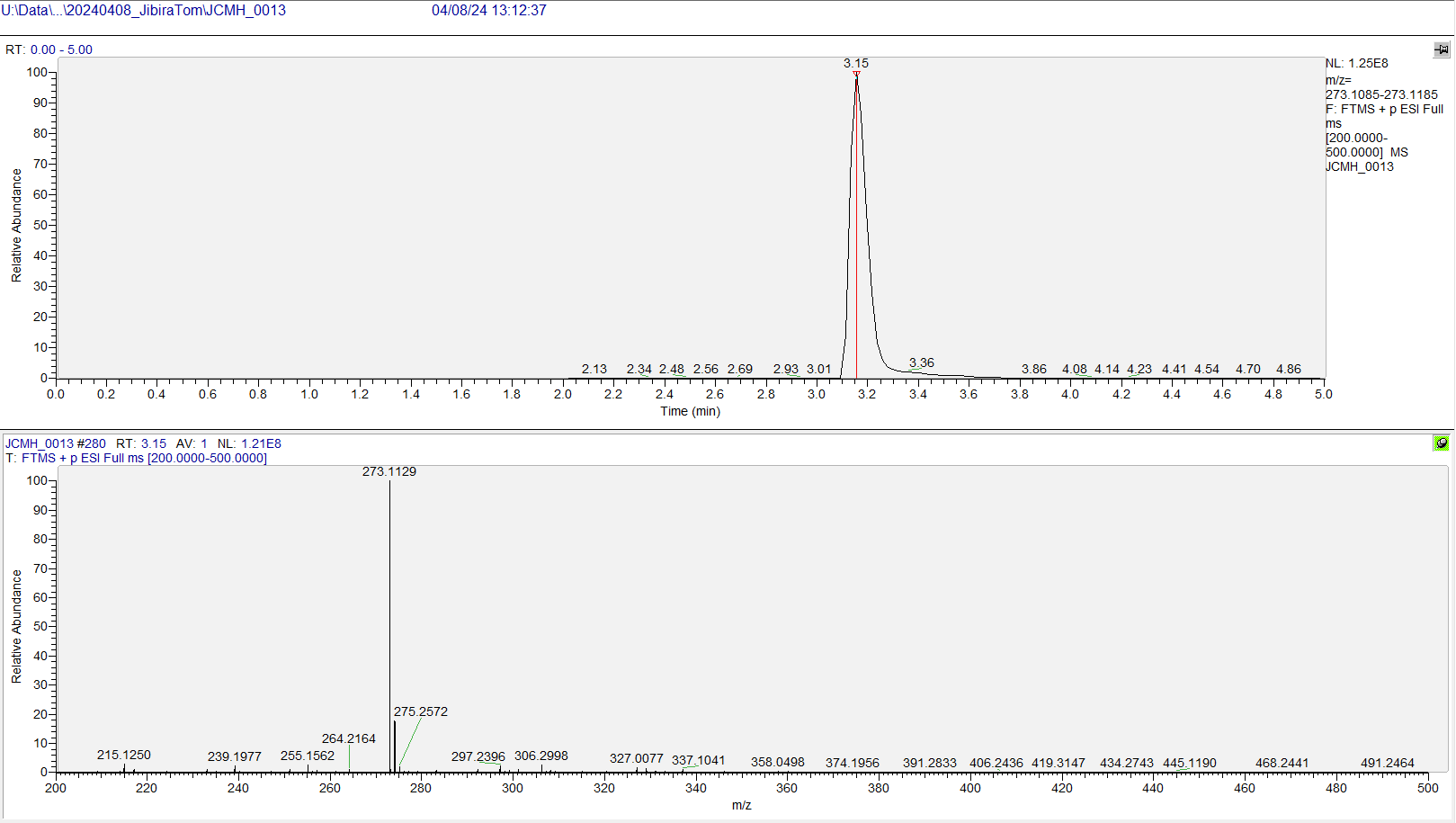


## Compound 4

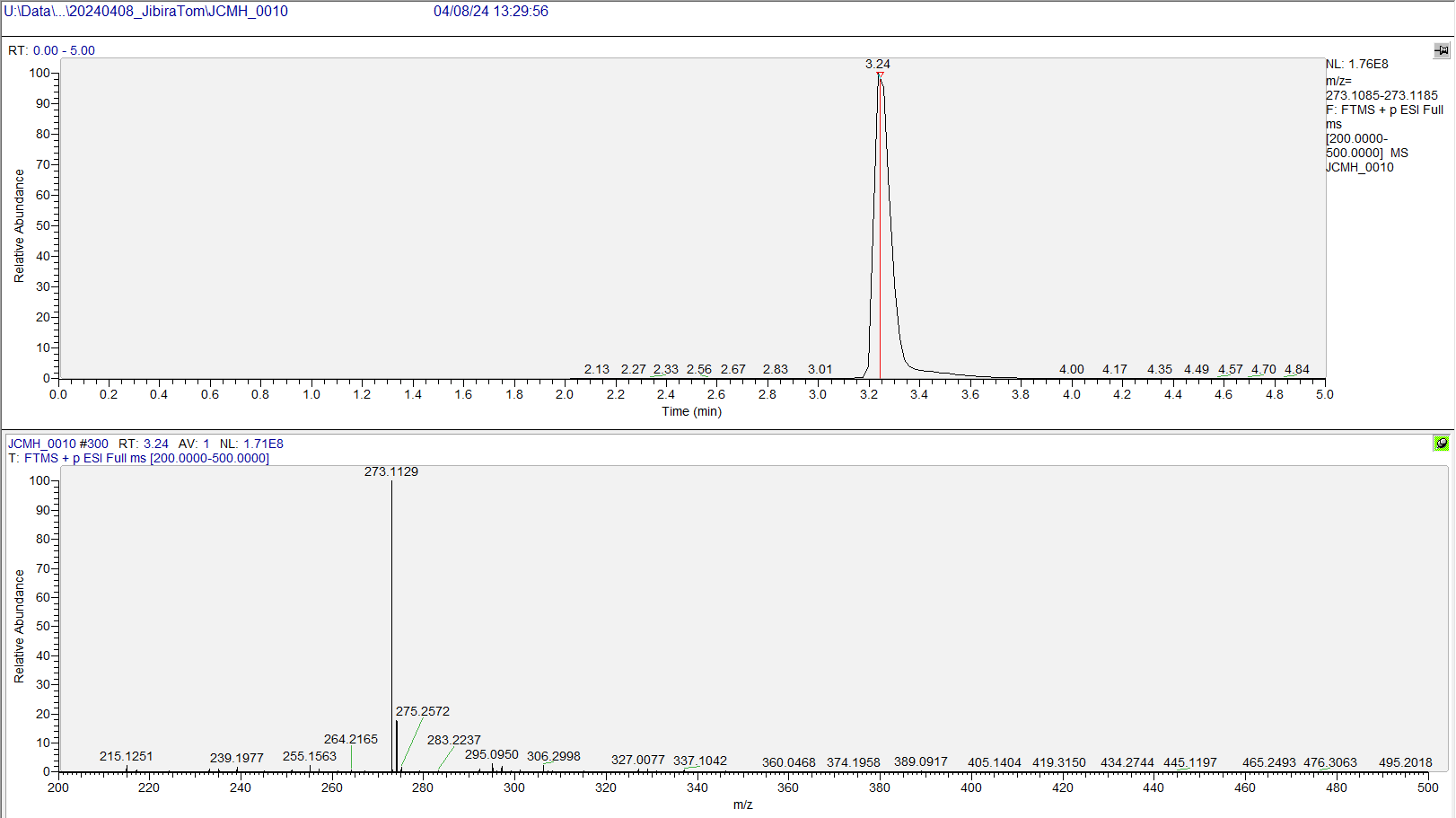


## Compound 5

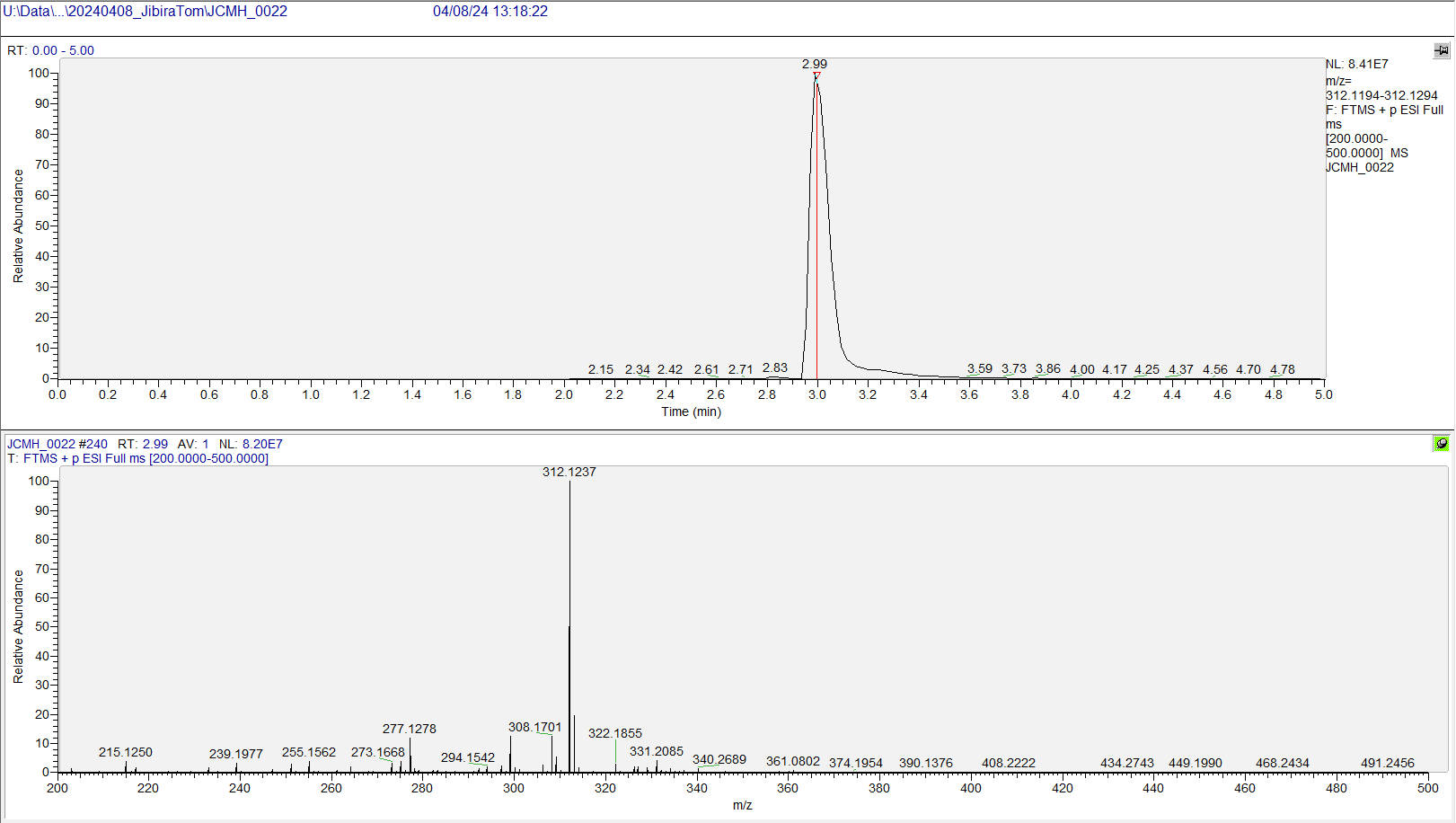


## Compound 6

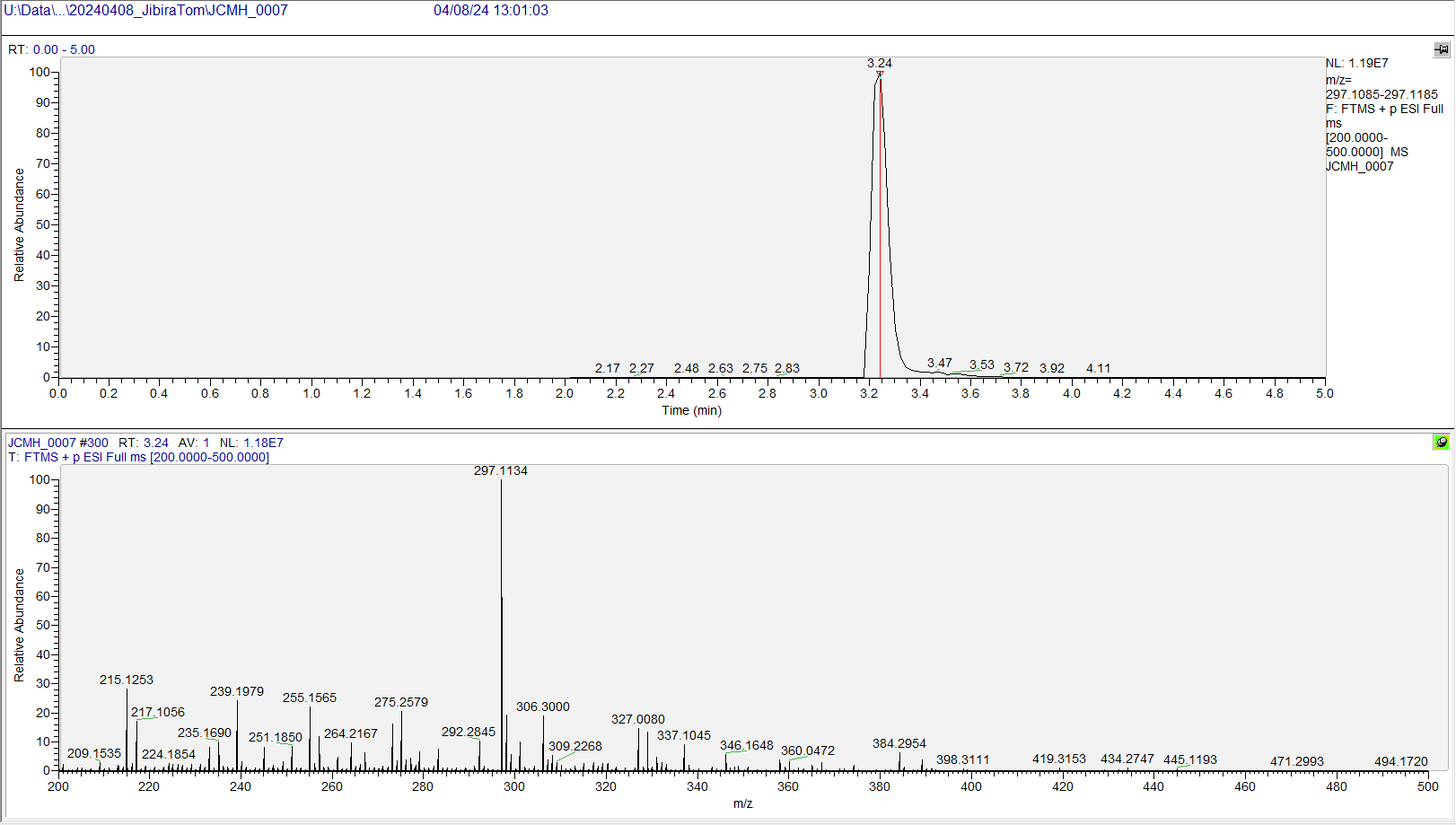


## Compound 7

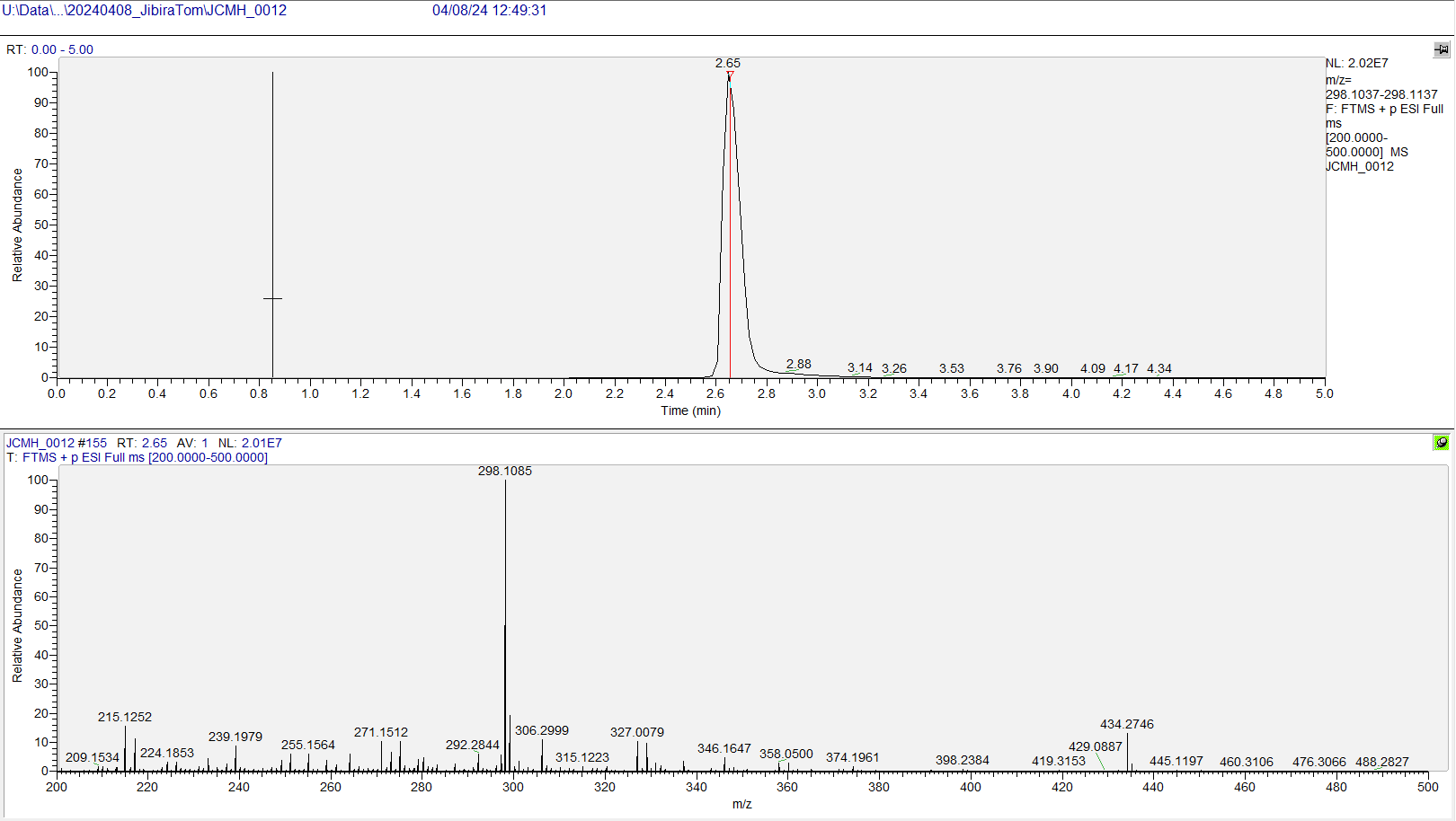


## Compound 8

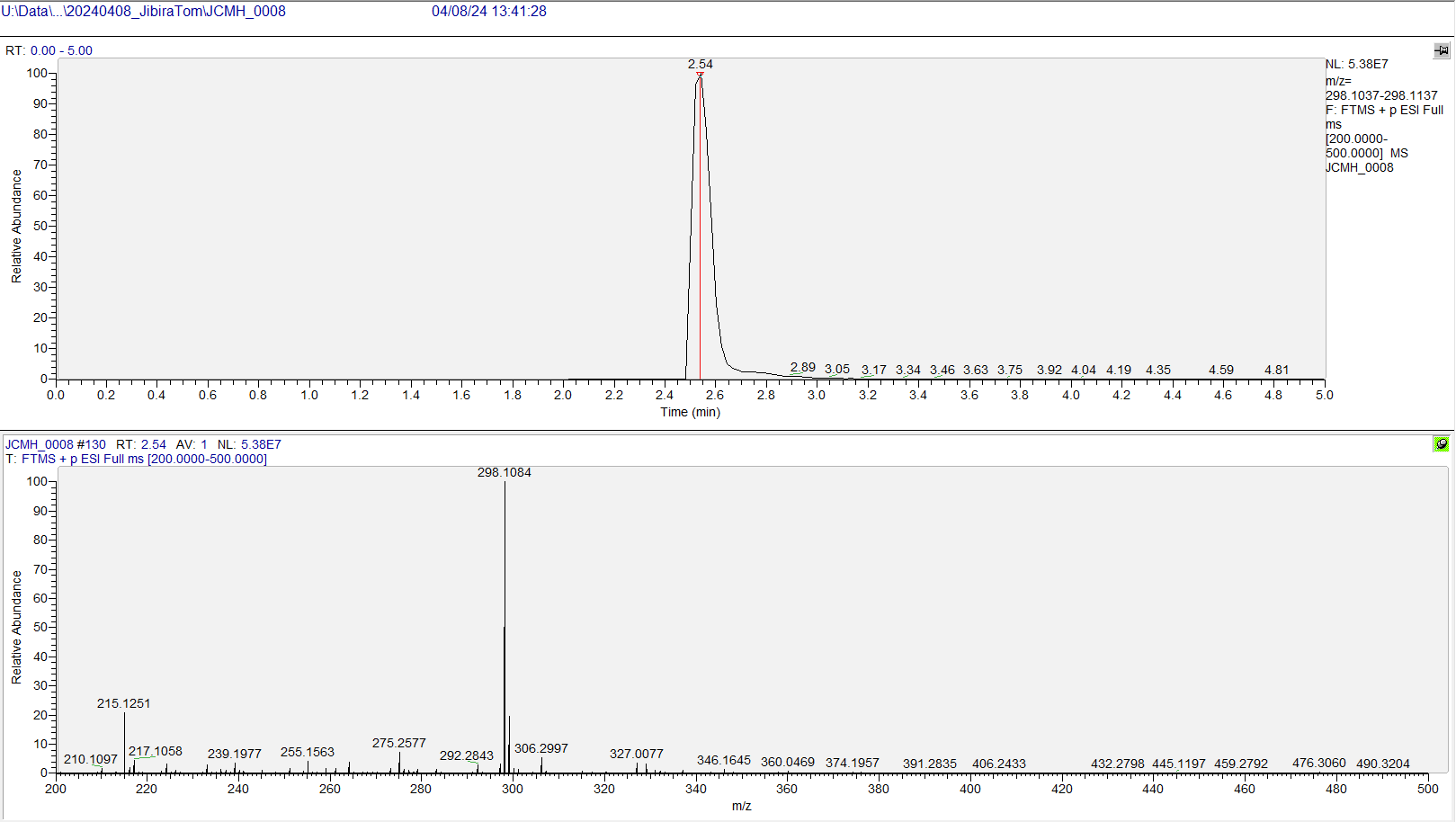


## Compound 9

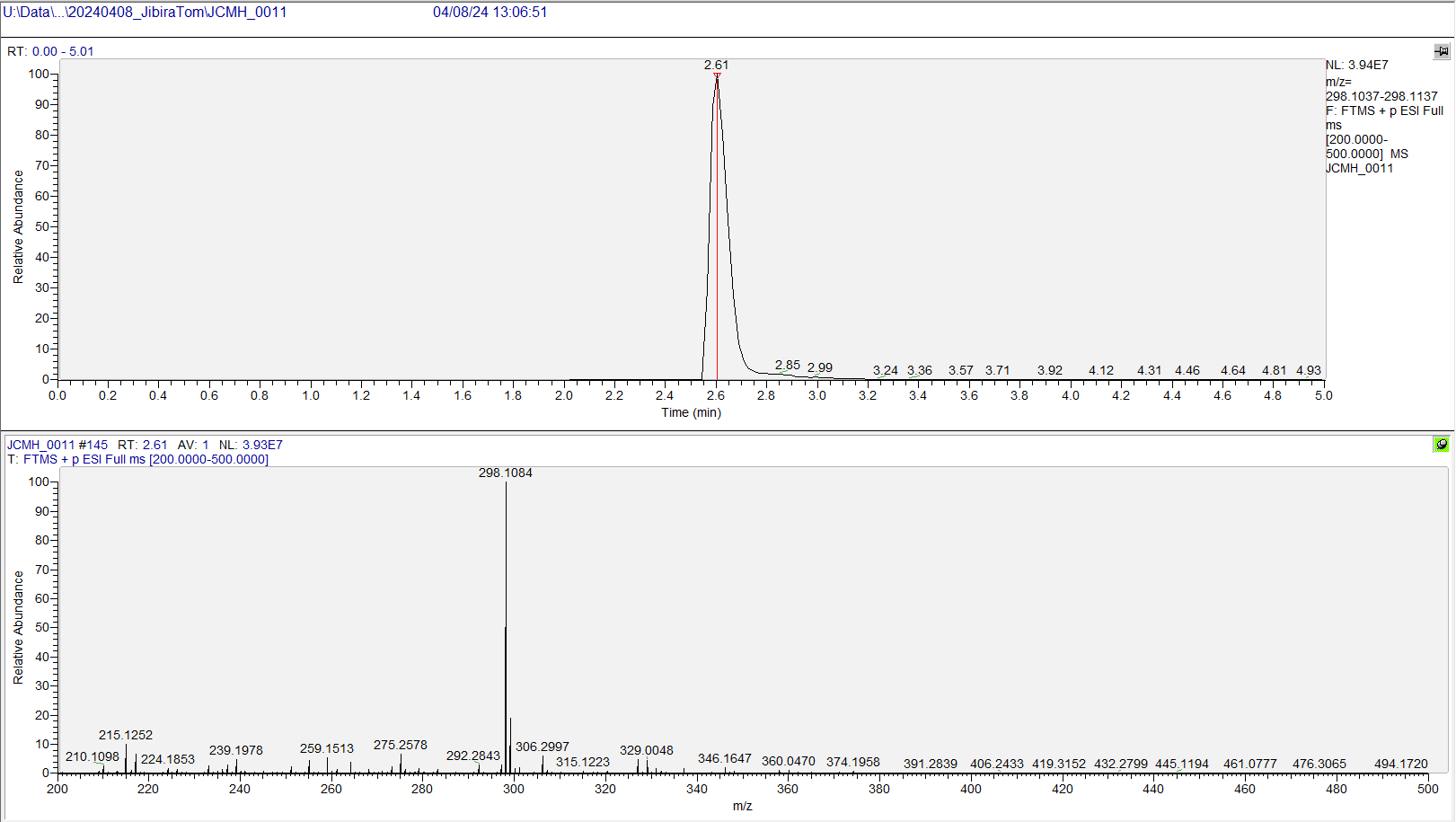


## Compound 10

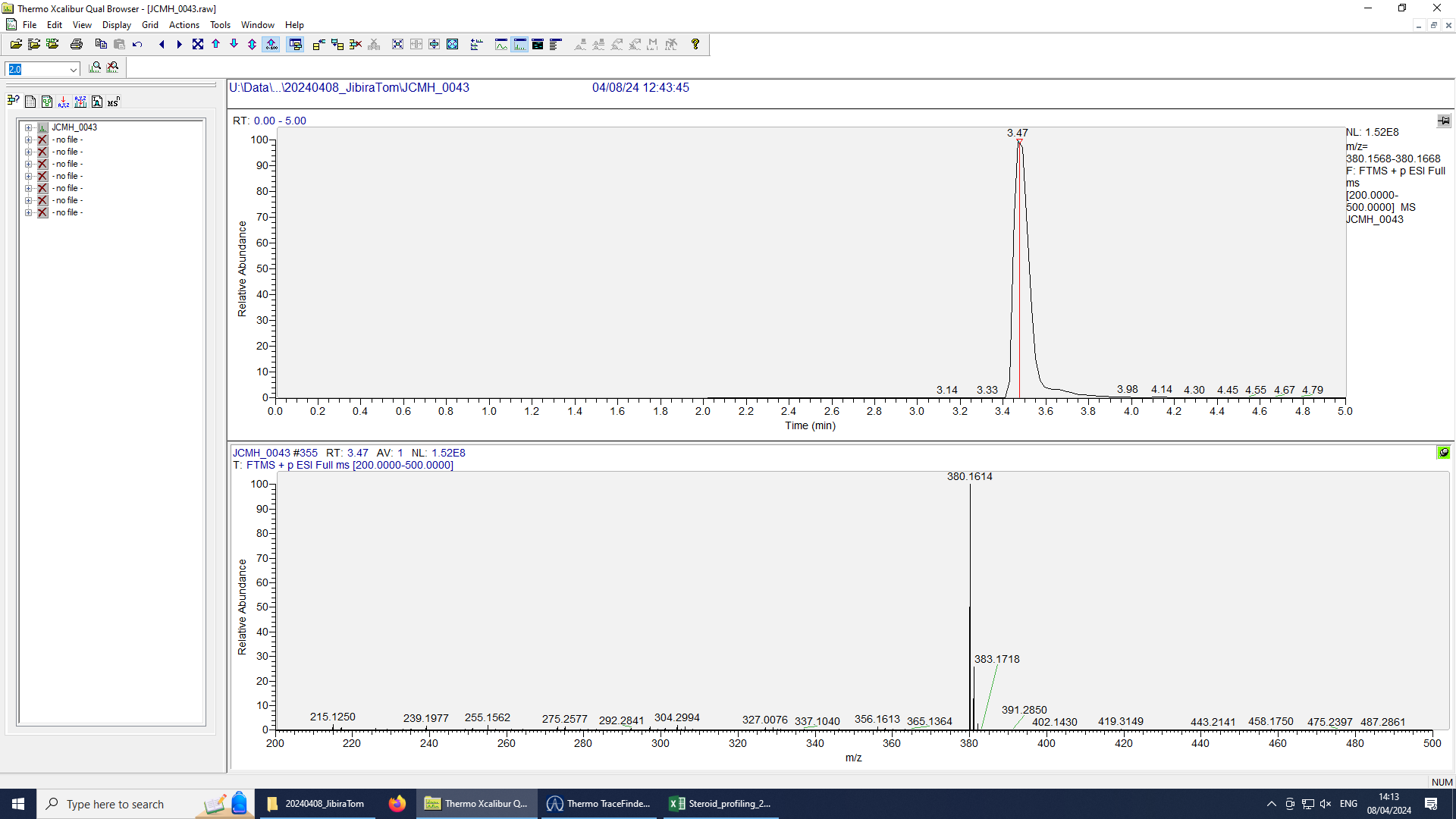


## Compound 11

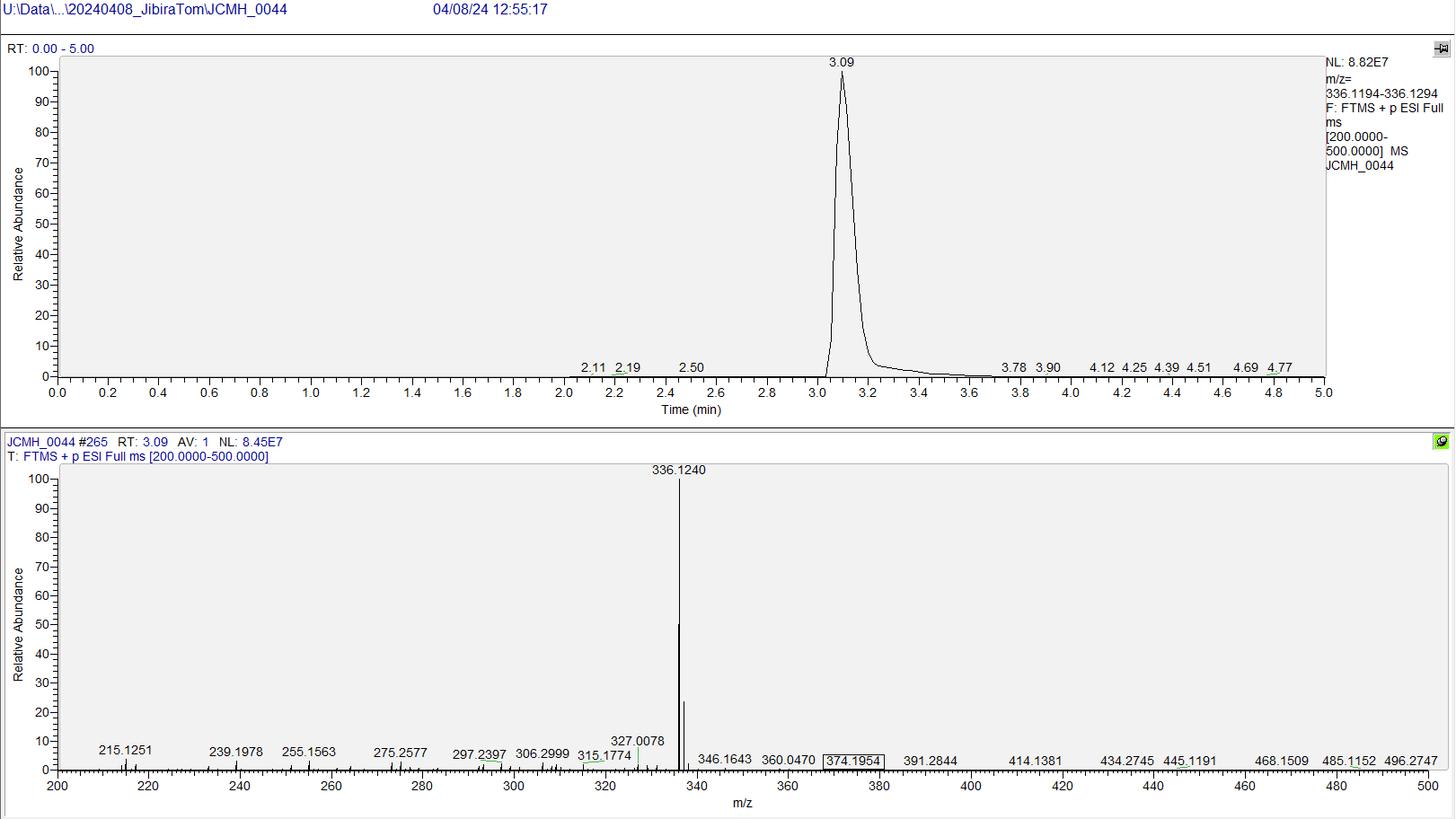


## Compound 12

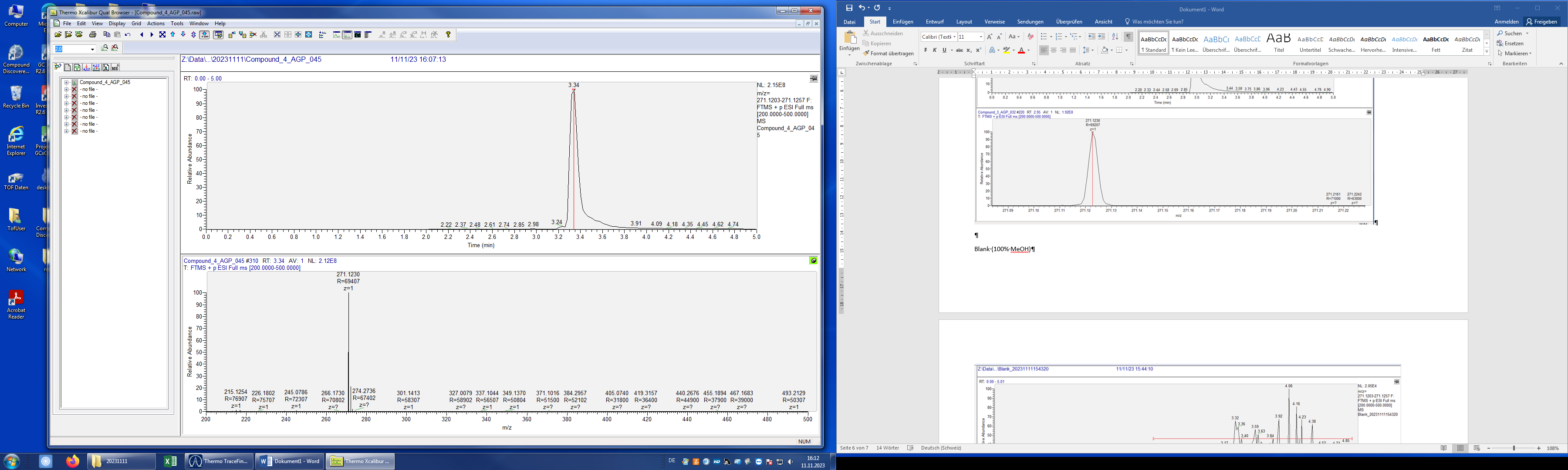


## Compound 13

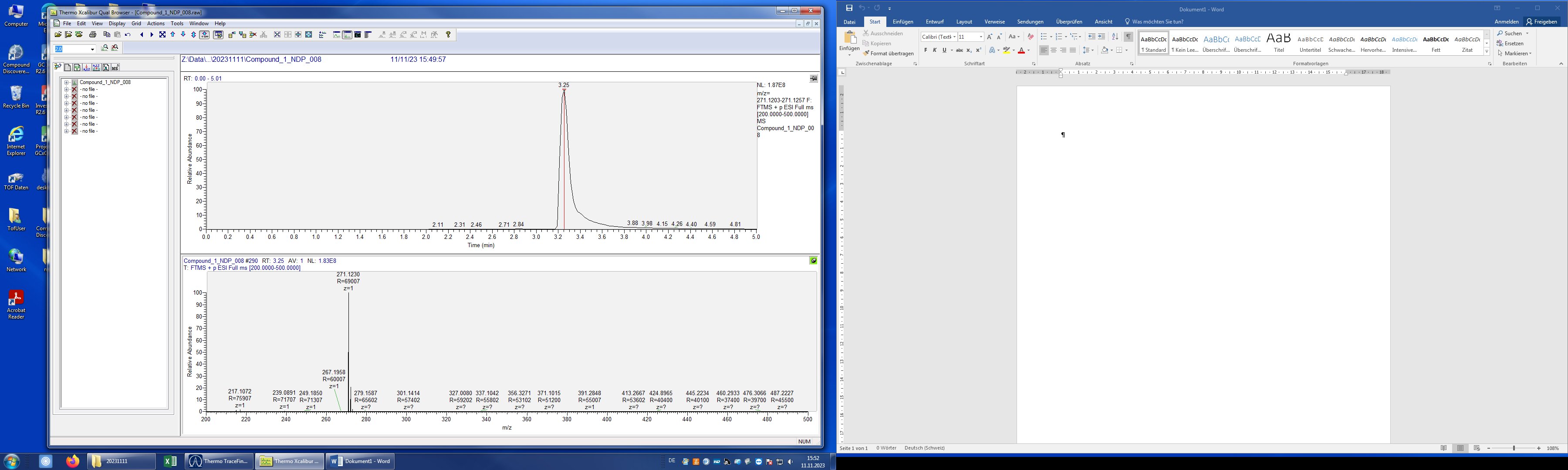


## Compound 14

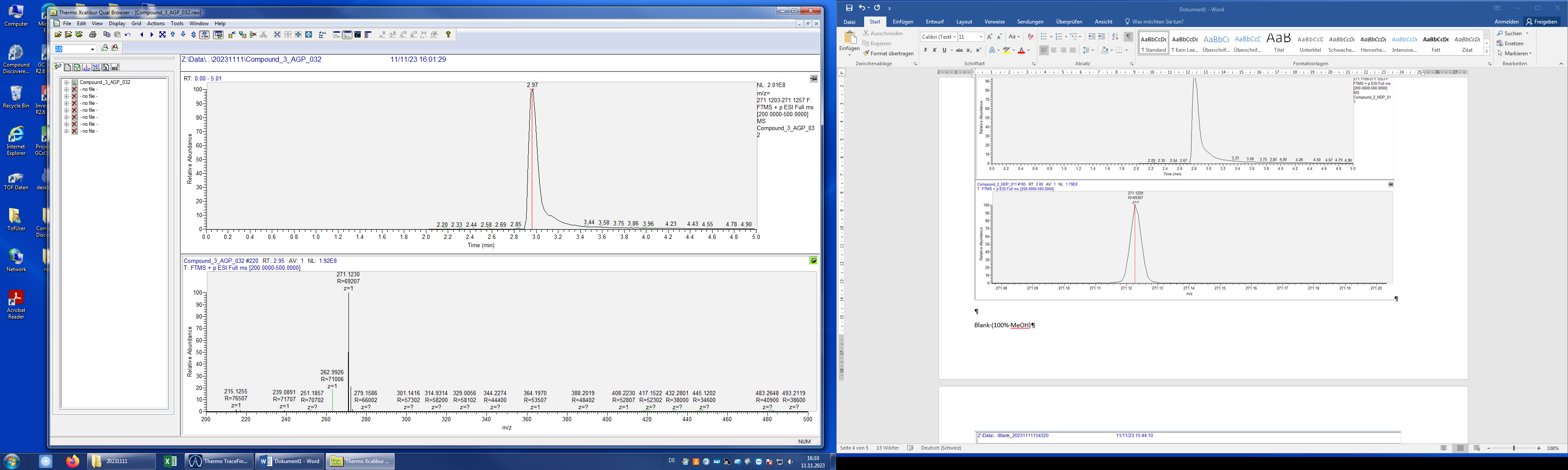


## Compound 15

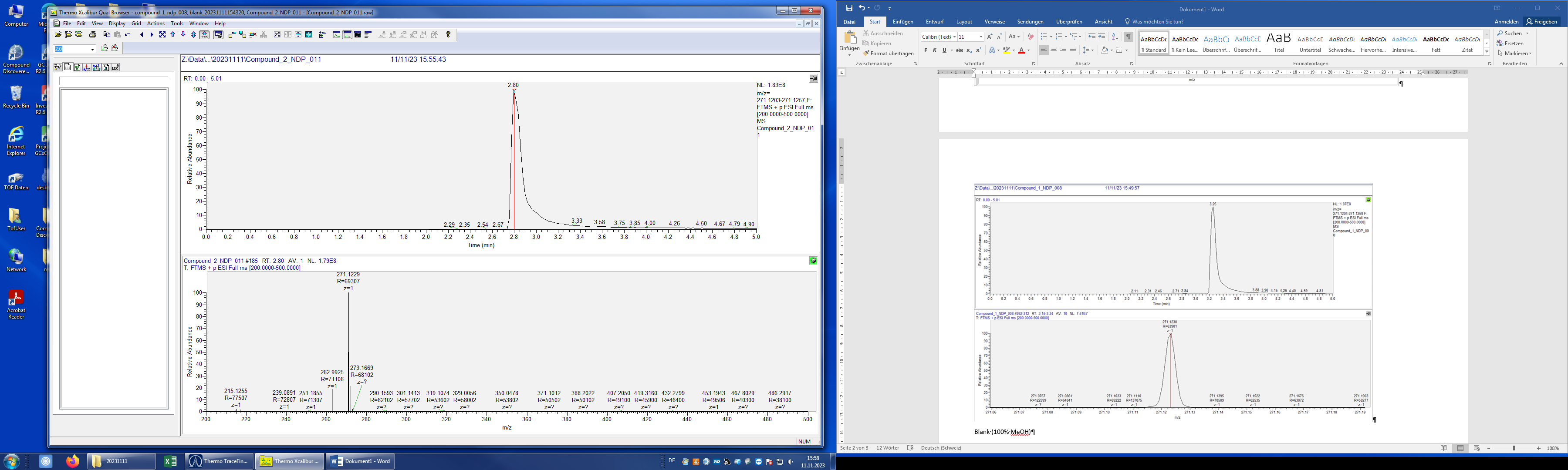

Supplement: supp_info_JEIMC_ Clean.docx [file IENZ_A_2463014_SM9512.docx]
